# Dr.VAE: improving drug response prediction via modeling of drug perturbation effects

**DOI:** 10.1093/bioinformatics/btz158

**Published:** 2019-03-08

**Authors:** Ladislav Rampášek, Daniel Hidru, Petr Smirnov, Benjamin Haibe-Kains, Anna Goldenberg

**Affiliations:** 1 Department of Computer Science, University of Toronto, Toronto, ON, Canada; 2 Genetics & Genome Biology, SickKids Research Institute, Toronto, ON, Canada; 3 Vector Institute, Toronto, ON, Canada; 4 Princess Margaret Cancer Centre, University Health Network, Toronto, ON, Canada; 5 Department of Medical Biophysics, University of Toronto, Toronto, ON, Canada

## Abstract

**Motivation:**

Individualized drug response prediction is a fundamental part of personalized medicine for cancer. Great effort has been made to discover biomarkers or to develop machine learning methods for accurate drug response prediction in cancers. Incorporating prior knowledge of biological systems into these methods is a promising avenue to improve prediction performance. High-throughput cell line assays of drug-induced transcriptomic perturbation effects are a prior knowledge that has not been fully incorporated into a drug response prediction model yet.

**Results:**

We introduce a unified probabilistic approach, Drug Response Variational Autoencoder (Dr.VAE), that simultaneously models both drug response in terms of viability and transcriptomic perturbations. Dr.VAE is a deep generative model based on variational autoencoders. Our experimental results showed Dr.VAE to do as well or outperform standard classification methods for 23 out of 26 tested Food and Drug Administration-approved drugs. In a series of ablation experiments we showed that the observed improvement of Dr.VAE can be credited to the incorporation of drug-induced perturbation effects with joint modeling of treatment sensitivity.

**Availability and implementation:**

Processed data and software implementation using PyTorch (Paszke *et al.*, 2017) are available at: https://github.com/rampasek/DrVAE.

**Supplementary information:**

Supplementary data are available at *Bioinformatics* online.

## 1 Introduction

Personalized drug response prediction promises to improve the therapy response rate in life-threatening diseases, such as cancer. There are two main impediments that make the task of drug response prediction highly challenging. First, the space of all possible treatments and their combinations for a given condition is prohibitively large to be explored exhaustively in clinical settings, drastically limiting the sample size for many therapies and tissues of interest. Second, cancer heterogeneity among patients is very high, reducing the statistical power of biomarker detection. These two conditions make it hard to characterize the genotype-to-phenotype landscape comprehensively making it difficult to accurately stratify drug treatment options for a particular cancer patient. To fulfill the promise of precision medicine, we need predictive models that can take advantage of heterogeneous, sparsely sampled data and data generated from pre-clinical model systems, such as cancer cell lines, to improve our prediction ability.

In the last decade there have been several public releases of large-scale drug screens in cancer cell lines. The greatest advantage of cell lines is their potential for high-throughput experiments as it is possible to screen cell lines against thousands of chemical compounds, both clinically-approved and experimental. This screening task was undertaken by several large consortia and pharmaceutical companies resulting in large, valuable public data resources (Barretina *et al.*, 2012; Garnett *et al.*, 2012; Haverty *et al.*, 2016; Rees *et al.*, 2016; Reinhold *et al.*, 2012; Yang *et al.*, 2013). The availability of these large cancer cell line datasets spurred the development of predictive models (Azuaje, 2017; Azuaje *et al.*, 2017; Lee *et al.*, 2018; Papillon-Cavanagh *et al.*, 2013; Safikhani *et al.*, 2017; Tan *et al.*, 2018; Wang *et al.*, 2017; Zhang *et al.*, 2015, 2018) and computational challenge-based competitions (Costello *et al.*, 2014; Menden *et al.*, 2018).

Papillon-Cavanagh *et al.* (2013) compared five feature selection approaches combined with linear regression modeling using the Genomics of Drug Sensitivity (Garnett *et al.*, 2012) dataset as training set and the Cancer Cell Line Encyclopedia (Barretina *et al.*, 2012) as independent validation set. They identified univariate and elastic net as the most robust approaches to develop predictors of drug response. They further improved their initial results by developing the minimum Redundancy, Maximum Relevance Ensemble feature selection (De Jay *et al.*, 2013). Jang *et al.* (2014), in a large methods evaluation effort, compared seven standard machine learning approaches, such as (sparse) linear models, random forest and support vector machines, for drug response prediction in the same Genomics of Drug Sensitivity and Cancer Cell Line Encyclopedia datasets. Their study identified ridge and elastic net regressions as the best performers. They and several other studies (Costello *et al.*, 2014; Stetson *et al.*, 2014), evaluated leveraging multi-omic data to enhance response predictors, generally demonstrating potential for performance improvement, but identifying gene expression as the single most informative data modality. Further, significant research has been done to explore ways to increase predictive power by additionally incorporating chemical features of drug compounds (Menden *et al.*, 2013; Wang *et al.*, 2017; Zhang *et al.*, 2015, 2018), or prior knowledge such as drug targets or biological networks (Azuaje *et al.*, 2017; Lee *et al.*, 2018).

Particularly influential has been the NCI-DREAM drug prediction challenge, presented in Costello *et al.* (2014). This challenge had 44 competing methodological submissions, categorized into six major methodological types. Their post-competition analysis revealed two particular trends among the most successful methods, the ability to model non-linear relationships between data and outcomes, and incorporating prior knowledge such as biological pathways. The winner of this challenge incorporated these approaches together with multi-drug learning by developing Bayesian multitask multiple kernel learning method (Costello *et al.*, 2014).

Complementary to large-scale cell line viability screens, the National Institutes of Health Library of Integrated Network-based Cellular Signatures (NIH LINCS) Connectivity Map (CMap) (Subramanian *et al.*, 2017) project measured the transcriptional perturbations induced by over 20 000 chemical compounds by profiling 1000 landmark genes in a set of 77 human cell lines before and after short-term drug treatment. These case-control matched experiments show how the expression of these genes changed in response to drug treatment at various concentration levels, typically after 6 or 24 h treatment duration. The set of drug-induced up- and down-regulation signatures is referred to as a drug perturbation signature (Smirnov *et al.*, 2015; Subramanian *et al.*, 2017). Combining response and perturbation data is expected to ultimately yield a better and more biologically relevant model of drug response (Niepel *et al.*, 2017; Subramanian *et al.*, 2017).

Previous work by Niepel *et al.* (2017) studied transcriptomic perturbations of six breast cancer cell lines, from an initial CMap release, in combination with phenotypic drug response measurements to determine whether cell lines that have similar phenotypic drug response also share common patterns in drug-induced gene expression perturbation. Their analysis concluded that this is the case for some drugs (inhibitors of cell-cycle kinases), but for other drugs the molecular response was cell-type specific, and for some drug-cell line combinations a significant transcription perturbation had no measurable impact on cell growth. These results motivated us to develop a unified method that could identify more complex associations of molecular perturbations and phenotypic responses that are potentially unique to a cell line subgroup.

The drug response prediction problem suffers from a high feature-to-sample ratio, where only a limited number of samples are available compared to the large number of measured molecular features (e.g. gene expression levels for thousands of genes). One way to alleviate this hindrance is to find a reduced representation of the original data that captures the essential information needed for the prediction task. Here, we take the approach of semi-supervised generative modeling based on variational autoencoders (VAE) (Kingma and Welling, 2014) that present a way to model complex conditional distributions. Way and Greene (2018) have shown that VAE can extract biologically meaningful representation of cancer transcriptomic profiles, while Dincer *et al.* (2018) combined a pre-trained VAE and a separately trained linear model in a drug response prediction method named DeepProfile. Contrary to Dincer *et al.* (2018) we aim to jointly learn a latent embedding that improves our ability to predict drug response (phenotypic outcome), while leveraging the originally unsupervised (unknown phenotypic outcome) drug perturbation experiments to aid in the learning of such embedding.

**Fig. 1.**
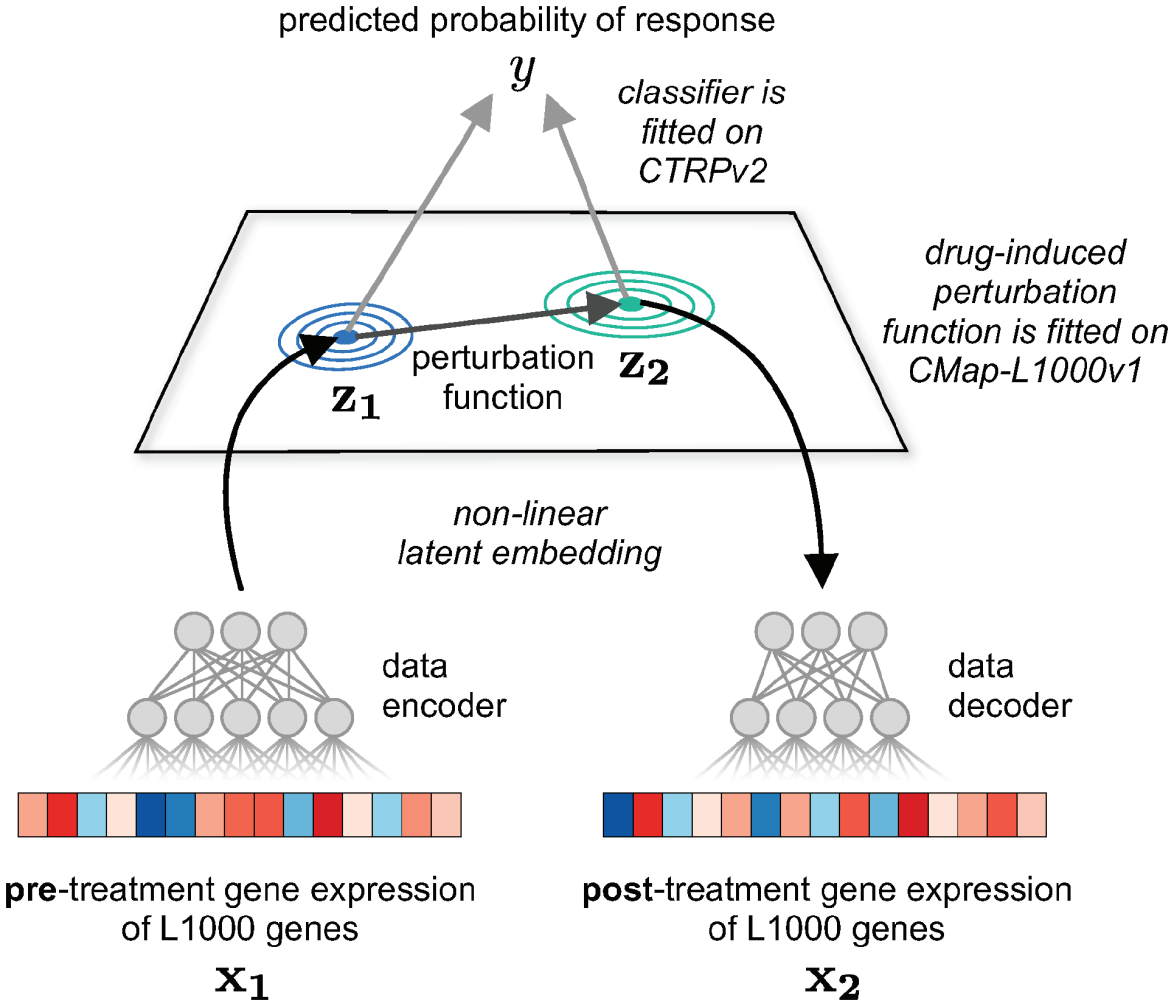
An overview of Dr.VAE prediction process. In training, Dr.VAE learns a drug response classifier jointly with a latent representation of pre-treatment gene expression and its drug-induced change. To make a prediction, we first embed the pre-treatment gene expression $x_{1}$, and then, from this latent representation $z_{1}$ we predict latent representation of post-treatment state $z_{2}$. Based on both $z_{1}$ and $z_{2}$, a logistic regression classifier predicts the probability of positive response. Additionally, we can decode the predicted post-treatment latent representation $z_{2}$ to the gene expression data space, but this is not required for drug response classification

We introduce Drug Response Variational Autoencoder (Dr.VAE), a deep generative model to predict drug response from transcriptomic perturbation signatures. Dr.VAE is a probabilistic graphical model where each conditional distribution is computed by a deep neural network. The model jointly learns a drug response predictor and a generative model of drug perturbation effects in a low-dimensional latent representation of gene expression. This latent space is defined by an encoder and decoder, both parametrized by a neural network, that, respectively, translate to and from this latent space. The entire model, together with neural networks for approximate inference, is optimized jointly end-to-end to maximize evidence (marginal likelihood) of the observed training data. An overview of Dr.VAE is illustrated in Figure 1.

In our results, Dr.VAE significantly outperformed classification models typically used in the field in more than half of the tested drugs and performed on par for most of the other drugs. We show that the achieved improvement of Dr.VAE in drug response prediction is indeed due to the joint modeling of drug response and drug-induced perturbation effects. This result is further confirmed by observing that even unsupervised generative modeling of gene expression and drug-induced perturbations yields a low-dimensional representation that is better suited for subsequent training of standard classification models than the original data representation or representation obtained by principal component analysis (PCA).

## 2 Materials and methods

### 2.1 Pharmacogenomics high-throughput cell line datasets

We harness two principally different types of pharmacogenomics datasets, both retrieved via PharmacoGx R package (Smirnov *et al.*, 2015) and PharmacoDB (Smirnov *et al.*, 2018). First is a database of sensitivity of cancer cell lines to drug treatment, the Cancer Therapeutic Response Portal (CTRPv2) (Rees *et al.*, 2016), that provides relative viability of cell lines at various drug concentration levels for combination of up to 860 cell lines and 481 drug compounds. Sensitivity of the cell lines to a drug treatment is quantified by the area above the dose-response curve (AAC), which was recomputed by PharmacoGx from raw CTRPv2 experimental results. We further binarized the continuous AAC by the waterfall method (Barretina *et al.*, 2012; Haibe-Kains *et al.*, 2013), turning the sensitivity prediction task into a discrete classification task.

Secondly, we utilized the NIH LINCS Consortium CMap project. The recently extended CMap, termed CMap-L1000v1 (Subramanian *et al.*, 2017), screened perturbation effects of 19 811 drug compounds on gene expression of L1000 landmark genes in up to 77 cell lines. Experiments in CMap-L1000v1 do not measure the drug treatment sensitivity, however some of the cell lines were independently tested in CTRPv2 as well. We cross-referenced these cell lines and assigned the corresponding label to their perturbation measurements.

From the CMap-L1000v1 dataset, we used the level 3 data, i.e. the quantile normalized gene expression of 978 landmark genes measured on Luminex based L1000 platform shown to be consistent with gene expression measured by RNAseq (Safikhani *et al.*, 2016; Subramanian *et al.*, 2017). From the available set of experimental conditions, we selected perturbation experiments with duration of 6 h conducted at the most common concentration level for each particular drug. That is, a concentration level that most cell lines were measured at for that drug. In case a cell line was not tested at the chosen concentration, we used the closest tested concentration. Next, we matched controls (DMSO vehicle) experiments to the drug perturbation experiments by the batch ID and bead ID, to minimize batch effects between the cases and controls. Further, we filtered the selected case-control pairs by correlation (>0.75 Pearson *ρ*) to filter out possibly mislabeled experiments or outliers.

CTRPv2 and CMap-L1000v1 datasets had 973 common genes. We standardized the expression values to zero mean and unit variance within each gene. For further homogenization, including batch effect removal and differences between two incorporated data sources, we also removed the first principal component (explaining 12.8% of variation) from the pooled dataset.

We selected 26 drugs tested in both CTRPv2 and CMap-L1000v1 datasets based on two simple criteria: (i) for each selected drug at least eight distinct cell lines were tested in CMap-L1000v1 perturbation experiments; and (ii) at least 20% of screened cell lines in CTRPv2 were sensitive to the drug after binarization of dose-response AAC. The dataset summary is detailed in Supplementary Table S6.

### 2.2 Dr.VAE

**Fig. 2.**
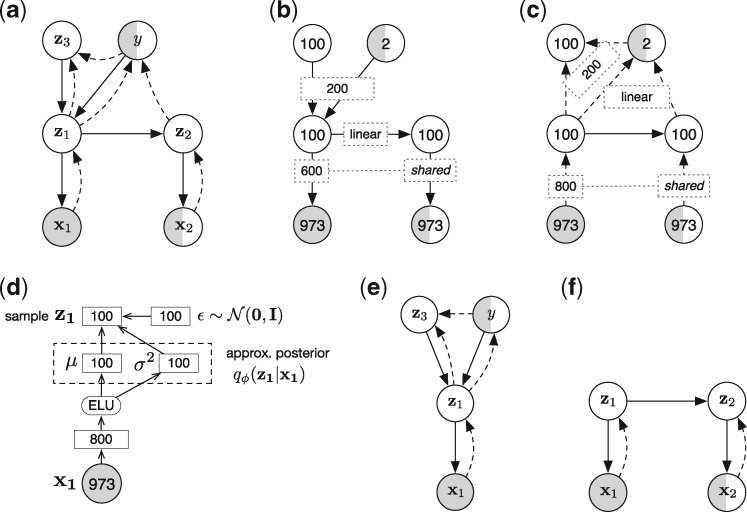
Dr.VAE model and its derivatives. (**a**) Factorization of the generative distribution *p* (solid edges) and of the approximate posterior *q* (dashed edges). In case the post-treatment gene expression $x_{2}$ is not observed, we use the expected posterior $E_{q_{\phi }(z_{1}|x_{1})}\left[p_{\theta }(z_{2}|z_{1})\right]$ for $z_{2}$ instead. (**b, c**) Hyperparameters of the generative and inference model, respectively. Node labels show dimensionality of the corresponding random variables, while edge labels show architecture of the encoders/decoders between the respective random variables. Note, that the ‘data decoder’ $p_{\theta }(x_{k}|z_{k})$ is shared for both k∈{1,2} and so is the ‘data encoder’ $q_{\phi }(z_{k}|x_{k})$. (**d**) Detailed depiction of data-to-latent-space encoder $q_{\phi }(z_{k}|x_{k})$ and of the reparameterization trick. (**e**) Factorization of SSVAE model (Kingma *et al.*, 2014), we set the hyperparameters of generative and inference distributions equivalently to the analogous distributions in Dr.VAE as shown in (b, c, d). (**f**) Factorization of PertVAE model, we set the hyperparameters of generative and inference distributions equivalently to the analogous distributions in Dr.VAE (b, c, d)

We present Dr.VAE, a new machine learning model based on a semi-supervised generative model. Dr.VAE learns a latent embedding of the gene expression. The latent embedding takes advantage of both cell line viability experiments that measure drug response outcome directly and, at the same time, the drug-induced transcription change, which in our case is modeled as a linear function in this latent space. This is achieved via joint training of the model on (i) ‘perturbation pairs’ $\left[x_{1},x_{2}\right]$ of pre-treatment (control) and post-treatment gene expression (outcome label y is only observed for some pairs) and (ii) ‘singletons’ of pre-treatment gene expression with no known post-treatment expression. Most of the outcome y labeled data are in the latter category. We model the drug perturbation effects with a single step latent time series model, similar to Deep Kalman Filter (Krishnan *et al.*, 2017) and structured graphical models with amortized inference (Johnson *et al.*, 2016). The graphical representation of Dr.VAE model is shown in Figure 2.

Formally, Drug Response VAE models a joint distribution $p(x_{1},x_{2},z_{1},z_{2},z_{3},y)$ of pre-treatment and post-treatment gene expression $x_{1},x_{2}$, their latent embedding $z_{1},z_{2}$, response class y, and class-independent latent representation of the pre-treatment expression $z_{3}$. Factorization of this joint probability distribution is depicted in Figure 2a (solid edges) and is as follows:

(1) $$p(x_{1},x_{2},z_{1},z_{2},z_{3},y)=$$

$$p(x_{1}|z_{1})\cdot p(x_{2}|z_{2})\cdot p(z_{2}|z_{1})\cdot p(z_{1}|z_{3},y)\cdot p(z_{3})\cdot p(y)$$

Individual conditional generative distributions p(·) of Dr.VAE take the form of diagonal multivariate Gaussian distributions, while p(y) is a uniform categorical prior over the binary response y and prior $p(z_{3})$ is a unit Gaussian N(0,I). The conditional distributions are parametrized by neural networks with a set of parameters *θ*, analogously to a VAE (Kingma and Welling, 2014; Rezende *et al.*, 2014). We want to model all gene expression measurements in a single latent space, thus the pre- and post-treatment gene expression have to be embedded into a common latent space. This is achieved by sharing the ‘data decoder’ $p_{\theta }(x_{k}|z_{k})$ for both k∈{1,2}. Additionally, we constrain the mean function of the perturbation $p_{\theta }(z_{2}|z_{1})$ to be a linear function $z_{1}+Wz_{1}+b$. Here, the **W** and **b** are initialized close to zero, such that $p_{\theta }(z_{2}|z_{1})$ starts as an identity function in the beginning of optimization process.

In order to train and use our model, we need to be able to perform efficient inference of the hidden variables from the observed variables. We turn to stochastic variational inference and introduce an approximation *q* to the true posterior. We assume this approximate posterior *q* to factorize as shown in Figure 2a (dashed edges). Akin to generative distributions *p* introduced above, the variational distributions are diagonal multivariate Gaussian distributions, with exception of $q_{\phi }(y|z_{1},z_{2})$, parametrized by neural networks with a set of parameters ϕ. The ‘data encoder’ $q_{\phi }(z_{k}|x_{k})$, detailed in Figure 2d, is shared between pre- and post-treatment for the same reason the data decoder is shared. The classification posterior $q_{\phi }(y|z_{1},z_{2})$ is a categorical distribution parametrized by a linear function with soft-max activation over two output units. In our implementation, we use the latent embedding of pre-treatment state and the predicted perturbation difference $\left[z_{1},z_{2}-z_{1}\right]$ instead of $\left[z_{1},z_{2}\right]$ as the classifier input. We found that this slightly improves the performance.

Ideally we would want to fit the *θ* and ϕ parameters to maximize the evidence (marginal likelihood) of the observed data, which is a difficult task and subject to active research in the area of stochastic inference. However, following Kingma *et al.* (2014); Kingma and Welling (2014) and Louizos *et al.* (2015) we can derive a lower bound on the evidence of each set of observed variables. We have four different sets of observed variables that correspond to four different types of data we want to fit Dr.VAE to. Therefore there are four different evidence lower bounds for us to optimize:

(2) $$\text{labeled}\;\text{perturbation}\;\text{pairs}\;\mathrm{LP}:\;\sum L_{\text{LP}}(x_{1},x_{2},y;\theta ,\phi )$$

(3) $$\text{unlabeled}\;\text{perturbation}\;\text{pairs}\;\mathrm{UP}:\;\sum L_{\text{UP}}(x_{1},x_{2};\theta ,\phi )$$

(4) $$\text{labeled}\;\text{pre}‐\text{treatment}\;\text{singletons}\;\mathrm{LS}:\;\sum L_{\text{LS}}(x_{1},y;\theta ,\phi )$$

(5) $$\text{unlabeled}\;\text{pre}‐\text{treatment}\;\text{singletons}\;\mathrm{US}:\;\sum L_{\text{US}}(x_{1};\theta ,\phi ).$$

The sum of these four specific evidence lower bounds, ${\text{ELBO}}_{\text{DrVAE}}$, is the evidence lower bound we need to maximize. Moreover, we need to explicitly introduce cross-entropy loss of the predictive posterior $\text{log}\;q_{\phi }(y|z_{1},z_{2})$ so that it is trained on labeled data as well. Analogous to semi-supervised variational autoencoder (SSVAE) (Kingma *et al.*, 2014), this explicit loss is required since in the labeled data the random variable y is observed and therefore the lower bounds $L_{\mathrm{LP}}$ and $L_{\mathrm{LS}}$ are conditioned on y and do not contribute to fitting of $q_{\phi }(y|z_{1},z_{2})$. Using the reparameterization trick (Kingma and Welling, 2014) it is possible to backpropagate through the final objective and jointly optimize parameters of all $p_{\theta }$ and $q_{\phi }$ distributions by gradient decent. In our implementation, we compute the parameter updates by Adam (Kingma and Ba, 2015) for both *θ* and ϕ parameters. Derivation of the final objective function is presented in Supplementary Material.

Detailed Dr.VAE architecture is shown in Figure 2b–d. Throughout the model, we used ELU activation function (Clevert *et al.*, 2015) as the non-linearity of our choice.

### 2.3 Perturbation variational autoencoder

We specifically denote the part of Dr.VAE that models drug-induced gene expression perturbations as the Perturbation Variational Autoencoder (PertVAE). PertVAE is an unsupervised model, depicted in Figure 2f, which we use to study the contribution of drug effect modeling on learned latent gene expression representation. We parameterize the PertVAE the same way as analogous parts in Dr.VAE. Detailed derivation of PertVAE is presented in Supplementary Material.

## 3 Results

We evaluated our drug response prediction method, Dr.VAE, on 26 Food and Drug Administration-approved drug compounds selected from the intersection of two independent in vitro drug screening studies: (i) the CTRPv2 (Rees *et al.*, 2016) where viability of up to 855 cell lines was measured in response to drug treatment, and (ii) drug-induced transcriptomic perturbations, assayed by NIH LINCS Consortium CMap project (CMap-L1000v1) (Subramanian *et al.*, 2017), in up to 60 different cell lines for the selected set of drugs.

We compared Dr.VAE to ridge logistic regression (RidgeLR), random forest (RForest) with 100 trees, and support vector machine with a radial basis function kernel (SVMrbf) applied directly to gene expression and also transformed through dimensionality reduction. We used the implementation of these methods as available in the scikit-learn library (Pedregosa *et al.*, 2011). For each drug, the best regularization parameter of RidgeLR was found in cross-validation. To assess the impact of drug-induced perturbations on the drug response prediction task we also compared Dr.VAE to SSVAE (Kingma *et al.*, 2014) where the focus is on classification using solely pre-treatment gene expression. SSVAE does not include any information of drug-induced transcriptomic perturbations. All evaluated models were fit independently to each of the 26 drugs, reusing the same deep learning architecture. We assessed the performance of the classifiers using the area under the ROC curve (AUROC) and the precision recall curve (AUPR) (presented in Supplementary Material).

We generated 100 train-validation-test data splits by performing repeated 5-fold cross-validation 20-times. The perturbation data from CMap-L1000v1 were split based on cell line identifiers so that all measurements pertaining to one cell line were assigned to one fold. The CTRPv2 sensitivity data were split such that the ratio of responders/non-responders was approximately equal in each fold, except cell lines that are in the intersection of CTRPv2 and CMap-L1000v1, which were assigned to their corresponding CMap-L1000v1 folds. The CMap-L1000v1 folds were pooled into training and validation splits only, as for some drugs the availability of perturbation experiments was limited to only as few as eight cell lines. Therefore test splits consisted exclusively of data from CTRPv2 that had no known post-treatment gene expression. This way Dr.VAE is most fairly evaluated against methods that cannot model perturbation effects, which is the typical scenario when response prediction has to be made solely based on pre-treatment features. During training of Dr.VAE and SSVAE models, a validation fold was used for early stopping and selection of classification loss weight. All compared methods were trained and evaluated on the same 100 train-validation-test data splits.

### 3.1 Drug response prediction from expression of L1000 genes

We jointly trained Dr.VAE on both CTRPv2 cell line sensitivity dataset and CMap-L1000v1 6 h-long perturbations and compared the performance to three established baseline classification models. Each model was trained on the expression of 973 genes that form the intersection of genes measured by the L1000 platform in CMap and RNAseq in CTRPv2. For a fair comparison, the baseline classifiers were trained on the very same data splits as Dr.VAE, consisting of CTRPv2 and CMap pre-treatment (control) experiments. Following the random variable notation from our Dr.VAE model, Figures 1 and 2, these data correspond to $x_{1}$.

**Fig. 3.**
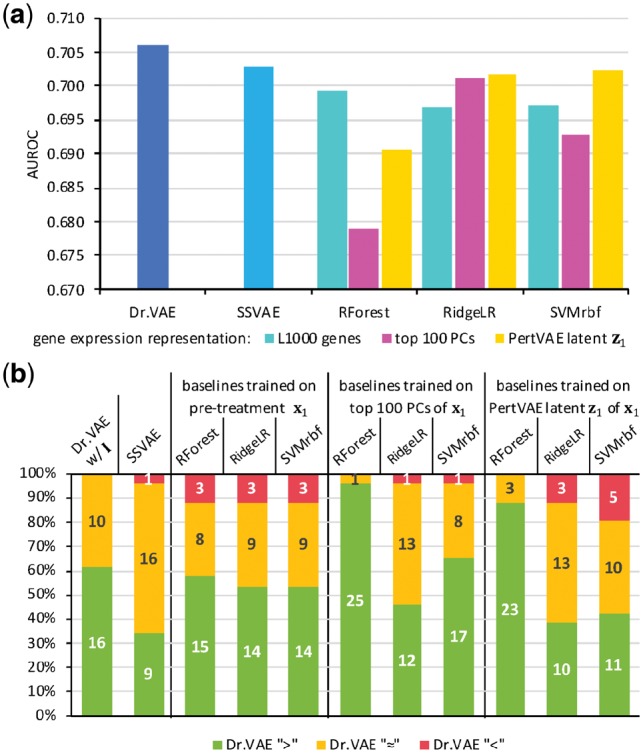
Summarized classification results. (**a**) AUROC of Dr.VAE and baseline methods. Shown is average over 26 drugs, each evaluated in 100 train-validation-test splits. (**b**) Dr.VAE is comparable or better than any other baseline for >80% of the drugs (*P*-value <0.05 Wilcoxon test)

**Fig. 4.**
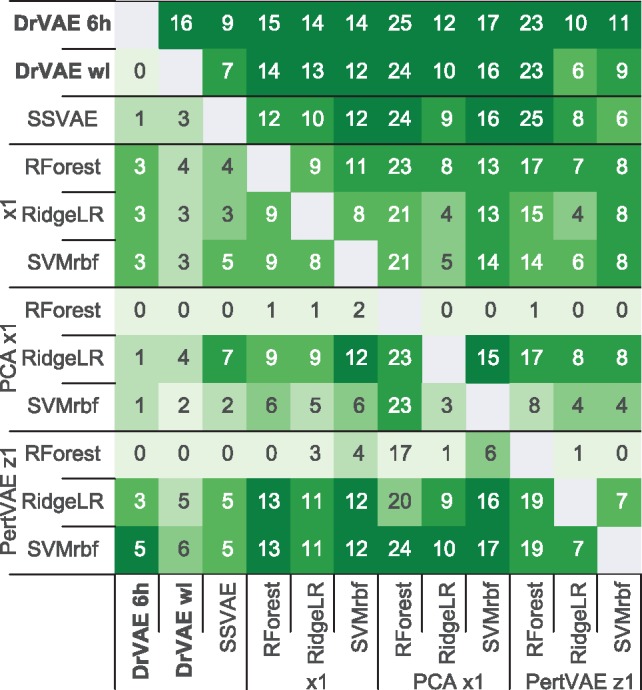
All to all comparison of tested methods. For each method, there is a row showing the count of 26 drugs for which this method significantly outperforms the other methods corresponding to individual columns. The comparison is based on test AUROC performance in 100 train-validation-test splits. Statistical significance of observed differences in test performance for any two methods was tested by one-sided Wilcoxon Signed-Rank Test (*P*-value <0.05). The heatmap color is normalized within each column, emphasizing methods that are the best contenders compared to the method corresponding to that column

Dr.VAE outperforms all three baseline classifiers for at least 14 out of 26 (53.8%) tested drugs, and performs with no statistically significant difference on nine drugs. On only 3 out of 26 (11.5%) drugs the baseline models performed better than Dr.VAE, Figures 3 and 4. The presented comparisons are based on one-sided Wilcoxon Signed-Rank Test (*P*-value <0.05) over 100 data splits. Detailed performance of all models applied to each individual drug is presented in Supplementary Table S1, the corresponding *P*-values are shown in Supplementary Table S2. Results in terms of the AUPR follow a similar pattern (Supplementary Material).

For bortezomib, niclosamide, paclitaxel, decitabine and clofarabine, cancer drugs with no established univariate biomarkers of response, Dr.VAE improved response prediction over every standard classification method by at least 1% and up to 4.4% of AUROC, while AUPR improved by at least 0.7% and up to 4.8%. We have observed the best improvement over RidgeLR for mitomycin and sirolimus with 5.7 and 3.9% AUROC improvement, respectively. Sirolimus inhibits the activation of a key regulatory kinase, the mammalian Target Of Rapamycin (mTOR). As showed in Niepel *et al.* (2017), perturbation effects induced by PI3K/Akt/mTOR kinases are typically cell-type specific, which possibly hampers response prediction for these drugs. In this case, Dr.VAE was able to better stratify the response classes, improving the response prediction, particularly over RidgeLR and SVMrbf. Mitomycin, an antibiotic that causes cross-linking of DNA and inhibition of DNA synthesis, is used as a chemotherapy drug in the treatment of various malignant neoplasms. Prediction of sensitivity to mitomycin treatment appears to benefit from employing non-linear prediction models such as RForest and SVMrbf. Dr.VAE can model non-linear relationships and performs on par with the RForest and SVMrbf, considerably outperforming RidgeLR.

Contrarily, in the case of fluvastatin and bosutinib, Dr.VAE trails RidgeLR by 1.5 and 0.9% in test AUROC, repsectively. Fluvastatin belongs to a class of drugs called statins. Statin inhibitors are used to control hypercholesterolemia but have been indicated to have a potential as anticancer agents as well. Sensitivity to statins is highly dependent on strength of a feedback mechanism, the activation of which has been reported to peak at time points >8 h post-treatment (Clendening *et al.*, 2010). Modeling of 6 h-long perturbations is insufficient in this case and as such Dr.VAE did not improve sensitivity prediction. Reduced performance of Dr.VAE in the case of bosutinib is likely due to modeling of perturbations at only the most common drug concentration level. Bosutinib is a tyrosine kinase inhibitor, used in chronic myelogenous leukemia therapy, primarily targeting Bcr-Abl kinase. Niepel *et al.* (2017) observed that such inhibitors of extracellular matrix receptors and receptor tyrosine kinases, exhibited considerably more variance in perturbation signatures with changing drug dose than other drugs. Since we selected perturbation experiments at only one drug concentration level, that with largest number of experiments, it is possible that modeling perturbation effects at only this one concentration level is not sufficiently informing the treatment sensitivity prediction.

### 3.2 Perturbation experiments improve drug response prediction

We investigated the contribution of drug perturbation experiments to response classification via two ablation studies. First, we compared Dr.VAE to semi-supervised VAE (Kingma *et al.*, 2014). SSVAE was fit to the pre-treatment gene expression in cell lines from CMap-L1000v1 and CTRPv2 without observing post-treatment gene expression and without modeling the drug effects. Since SSVAE is conceptually a subset of Dr.VAE’s architecture, we used the same hyperparameters for the corresponding encoders/decoders as in Dr.VAE, Figure 2e. SSVAE outperforms baseline methods according to AUROC but is not as good as Dr.VAE. Dr.VAE achieves significantly better test AUROC than SSVAE on 9 out of 26 (34.6%) drugs (*P*-value <0.05) with no statistically significant difference on 16 drugs (61.5%) and only for one drug (vincristine) SSVAE outperforms Dr.VAE, Figure 3.

To evaluate the contribution of the perturbation function to the classification performance, we modified each trained Dr.VAE instance by replacing the learned drug perturbation function with an identity function (denoted as ‘Dr.VAE w/I’) without retraining the rest of the model. The modified ‘Dr.VAE w/I’ achieves AUROC close to Dr.VAE, however slightly worse in absolute value over the 26 drugs. For 16 drugs Dr.VAE has significantly better performance than Dr.VAE w/I and for 10 drugs there was no significant difference, showing that while functions more complex than identity may be able to learn from the perturbation data, more drug perturbation data are required to substantially improve response prediction for many drugs.

Our results show that Dr.VAE improves drug response classification performance thanks to modeling of drug perturbation pairs. As our second set of experiments show, the learned perturbation function contributes to better classification. However, most of the observed improvement appears to stem from more informative latent gene expression representation, that, compared to SSVAE, is learned by joint modeling of drug perturbations as well as sensitivity response. The superior performance of Dr.VAE w/I compared to SSVAE is a testament to that effect.

### 3.3 The importance of dimensionality reduction

Dr.VAE and SSVAE learn a lower dimensional latent representation of the data and the classifier jointly. To understand the importance of the joint optimization, we also explored a learning paradigm where we first optimize the latent representation in an unsupervised fashion and only then train a classifier using the already learned embedding. To this end we performed two sets of experiments. First, we evaluated dimensionality reduction by PCA. PCA projects the data into a space given by orthogonal vectors called principal components that are selected in the order of largest possible variance they account for in the data. We chose to represent the CTRPv2 and CMap-L1000v1 pre-treatment gene expression of L1000 genes in terms of their first 100 principal components that we estimated on each training data fold. Second, we trained just the perturbation part of Dr.VAE, which we denote as PertVAE, to assess dimensionality reduction using a deep generative model. PertVAE is an unsupervised model that does not model drug response outcomes. Instead it learns to model drug perturbation effects from the perturbation pairs, Figure 2f. We then used the mean of the 100-dimensional latent embedding $z_{1}$ of the pre-treatment gene expression as the reduced representation for subsequent fitting of standard classifiers.

Both PCA and PertVAE were fit on each training data fold and the learned projections then applied to test data fold. We used the same 100 train-validation-test splits as in the previous experiments, thus the classification test results can be mutually compared by Wilcoxon Signed-Rank Test with the above mentioned Dr.VAE and multiple baseline results, Figures 3b and 4. In terms of mean AUROC, Figure 3a, and mean AUPR, Supplementary Figure S1, all three standard classifiers perform better when fit on the PertVAE embedding $z_{1}$ than when fit on the PCA projection onto the first 100 principal components. In the case of both of these reduced representations, notable is the improvement of the RidgeLR classifier that performs better than when trained directly on expression of the L1000 genes. These two methods, together with SVMrbf trained on the PertVAE $z_{1}$ embedding, achieve the most competitive results, nearly equal to SSVAE. However, our Dr.VAE model that combines PertVAE and a drug response classifier in an end-to-end fashion delivers the best overall classification performance, accomplishing statistically better or equivalent AUROC for at least 21 out of 26 drugs (80.8%) than any other evaluated method.

### 3.4 Modeling of drug perturbation effects

We have shown that Dr.VAE can distill useful information from drug perturbation experiments to improve cell line response classification. We seek to investigate how well Dr.VAE model can predict the actual post-treatment gene expression levels. In the following set of experiments we assessed how well Dr.VAE can predict the post-treatment expression in the latent space, corresponding to random variable $z_{2}$, as well as in the gene space, which corresponds to $x_{2}$. Particularly, we computed the expected root mean square error (RMSE) of Dr.VAE predictions over $z_{2}$ and $x_{2}$ when computed from pre-treatment $x_{1}$ compared to the expected embedding $z_{2}$ computed from post-treatment $x_{2}$ and the true observed $x_{2}$, respectively. Furthermore, we compared how the RMSE of Dr.VAE predictions improved over the ‘Dr.VAE w/I’ baseline model where we replaced the learned perturbation function by an identity function (as introduced previously). On training data, Dr.VAE predicted the mean of $z_{2}$ with RMSE 10.5% lower compared to Dr.VAE w/I, yet on validation data it was 9.6% worse on average across all 26 drugs. This result shows that Dr.VAE, while being primarily optimized for drug response classification, learns to partially model drug perturbation effects, but on average, suffers from data limitations and overfitting.

To elucidate the connection between Dr.VAE performance and limitations of available perturbation experiments, we computed the correlation of Dr.VAE $z_{2}$ prediction improvement over Dr.VAE w/I across the set of 26 drugs with three data statistics: (i) effect-to-replicate variance ratio (ERVR) in CMap-L1000v1 perturbation experiments, (ii) number of unique cell lines tested for a given drug in CMap-L1000v1 and (iii) the product of the previous two. The computed Pearson correlations are shown in Table 1. The ability of Dr.VAE to generalize from the training to validation set correlates with both the strength of the perturbation signal in the data (quantified as ERVR) and the dataset size, yet the strongest is correlation with the product of these two variables, ρ=0.814 (*P*-value $4.35\times 10^{-7}$). The computation of ERVR measure is described in Supplementary Material.

**Table 1.** The ability of Dr.VAE to model post-treatment gene expression correlates with signal/noise ratio and quantity of perturbation experiments

Δ RMSE evaluated on	dataset property correlated to	*ρ*	*P*-value
$z_{2}$	Effect/rep. variance ratio (ERVR)	0.66	$2.4\times 10^{-4}$
$x_{2}$	ERVR	0.72	$4.0\times 10^{-5}$
$z_{2}$	Num. unique CLs in CMap (NCL)	0.71	$4.2\times 10^{-5}$
$x_{2}$	NCL	0.52	$6.4\times 10^{-3}$
$z_{2}$	ERVR * NCL	0.81	$4.4\times 10^{-7}$
$x_{2}$	ERVR * NCL	0.73	$2.6\times 10^{-5}$
$x_{2}$	Dr.VAE-SSVAE [AUROC]	0.29	0.15
$x_{2}$	Dr.VAE-SSVAE [AUPR]	0.20	0.33

*Note*: We computed Δ RMSE improvement of Dr.VAE in post-treatment expression prediction over Dr.VAE w/I, averaged over validation data splits, and correlated it to overall CMap-L1000v1 dataset statistics. The Pearson correlation was computed for prediction Δ improvement of both post-treatment gene expression $x_{2}$ and its latent representation $z_{2}$. Additionally we include correlation with difference in Dr.VAE and SSVAE classification performance.

For prediction of post-treatment gene expression $x_{2}$ we observed an analogous conclusion to prediction of its latent representation $z_{2}$. The detailed results are shown in Supplementary Table S7. We conclude that there are presently data limitations (number and noise/signal resolution of drug perturbation experiments) for generalizable post-treatment gene expression prediction yet, as shown above, we can still distill information that improves drug response classification.

Lastly, we investigated whether there is a correlation between classification performance improvement of Dr.VAE over SSVAE, which does not model perturbation effects, and the ability of Dr.VAE to generalize post-treatment gene expression prediction to validation set. We found weak correlation between the classification improvement in terms of both AUROC (Pearson ρ=0.293; *P*-value 0.147), and AUPR (Pearson ρ=0.199; *P*-value 0.329). These results suggest that Dr.VAE tends to improve over SSVAE for the drugs Dr.VAE manages to model the transcriptomic perturbations induced by the drug compound.

## 4 Discussion

We developed Dr.VAE, the first unified machine learning method for drug response prediction that enables semi-supervised learning and successfully leverages prior information in the form of drug-induced transcriptomic perturbations. Our approach follows several previously identified trends for improved drug response prediction (Costello *et al.*, 2014), as we can model non-linearities in the data and incorporate prior knowledge.

Typical discriminative feedforward neural networks do not fare well in drug response prediction, most likely because of the data limitation (number of features versus number of samples). We showed that joint generative modeling of drug response and perturbation effects alleviates this to a significant extent, possibly acting as an effective regularization and robust feature extraction that does not overfit the way discriminative neural networks do.

We tested 26 Food and Drug Administration-approved drug compounds for which both perturbation and drug response experimental data were available. Our experiments showed that for those drugs that have sufficient data to capture the variation and effect on gene expression, incorporating those effects yields a significant improvement over logistic regression, random forest and support vector machines. Dr.VAE significantly outperformed these models in more than half of the tested drugs and performed on par in other cases. Through a series of experiments, we showed that the observed improvement of Dr.VAE in drug response prediction can be credited to its joint modeling of both response and drug-induced perturbation effects.

Our study has several potential limitations. First, we considered only the gene expression modality, as it has been consistently shown to provide the most predictive power in multiple previous studies on drug response (Costello *et al.*, 2014; Jang *et al.*, 2014). There is accumulating evidence, however, that multi-omic predictors that additionally integrate methylation, copy number variation, mutational status or proteomic data can achieve improved prediction performance. It is relatively straightforward to extend Dr.VAE, thanks to the stochastic variational inference approach we adopted. Categorical or Poisson likelihood functions can be used to model discrete (mutational status) or count (CNVs) data types, respectively, in addition to the Gaussian likelihood we used to model continuous gene expression. We caution however, that inclusion of additional features accentuates the already unfavorable ratio of the number of features to the number of available training examples, which could prove, and indeed has been, problematic for any method, including ours.

Second, we modeled CMap-L1000v1 perturbations after 6 h of treatment duration at the most common concentration level for each drug. That allowed us to pool the largest possible number of experiments tested under consistent experimental settings. It can be argued that 6 h is too short for many feedback regulatory mechanisms to manifest themselves and as such these experiments alone do not provide complete picture of the transcriptomic response. Notably, drug-cell line viability assays are typically done with longer treatment duration, such as 72 h. This is the case for a statin inhibitor fluvastatin, as we observed in out experiments. Thus we also trained our Dr.VAE with 24 h perturbation experiments, however, potentially because of the limited number of such experiments, this did not improve our prediction performance. A potential future improvement to our method could be an extension which leverages all available perturbation experiments of various durations and drug concentrations.

Every conditional distribution that Dr.VAE is composed of is parameterized by a neural network. The ability to adjust hyperparameters to match complexity of the data makes Dr.VAE a very flexible model. Since we opted for simplicity, most of our neural networks have one hidden layer, while the classification posterior and perturbation function are linear. As more data become available we will be able to take full advantage of the new methodological developments in the generative deep learning field, further improving the performance of Dr.VAE and other drug response prediction models. However so far our attempts to use deeper networks or utilize normalizing flows to approximate posteriors by more complex distributions (Kingma *et al.*, 2016; Rezende and Mohamed, 2015) have not significantly improved the performance to justify the added complexity.

## 5 Conclusion

In conclusion, we have demonstrated deep generative modeling to be a promising methodological approach for method development in the field of drug response prediction. In particular, this approach has two major benefits. First, the flexibility of this paradigm allowed us to integrate transcriptional perturbation effects into the drug response prediction framework in a unique way. Second, all conditional distributions that form our Dr.VAE model, as well as variational posteriors used for approximate inference in Dr.VAE, are parametrized by neural networks that can model complex non-linear relationships. We have shown that both aspects compounded in our Dr.VAE, which outperformed the most used methods in the field for the majority of the evaluated drug compounds.

## Funding

This work was supported by Canadian Cancer Society Research Institute Innovation Grant [703471]; the Natural Science and Engineering Research Council of Canada and the Canadian Institute for Health Research collaborative Health Research Project. The authors thank University of Toronto, SickKids Foundation, Cancer Research Society and the Gattuso Slaight Personalized Cancer Medicine Fund at Princess Margaret Cancer Centre for their generous funding.

*Conflict of Interest*: none declared.

## Supplementary Material

btz158_Supplementary_Material
