# Supplementary material for: Dr.VAE: improving drug response prediction via modeling of drug perturbation effects
Source: Bioinformatics. 2019 Mar 8;35(19):3743–51. doi: 10.1093/bioinformatics/btz158 (PMC6761940; doi:10.1093/bioinformatics/btz158)
Supplement: btz158_Supplementary_Material [file btz158_supplementary_material.pdf]

Supplementary Material

1 Method Details **1**

1.1 Perturbation variational autoencoder 1

1.2 Drug response variational autoencoder 3

2 Supplementary Results **7**

2.1 Effect-to-replicate variance ratio in perturbation experiments 7

2.2 Reconstruction of gene expression from latent representation 7

3 Supplementary Figures and Tables **9**

Method Details

1.1 Perturbation variational autoencoder

Perturbation Variational Autoencoder (PertVAE) is an unsupervised model for drug-induced gene expression perturbations, that embeds the data space (gene expression) in a lower dimensional latent space. In the latent space we model the drug-induced effect as a linear function, which is trained jointly with the embedding encoder and decoder.

We fit PertVAE on "perturbation pairs" $[\mathbf{x}_1, \mathbf{x}_2]$ of pre-treatment and post-treatment gene expression with shared stochastic embedding encoder $q_{\phi_{\mathbf{x}\to\mathbf{z}}}$ and decoder $p_{\theta_{\mathbf{z}\to\mathbf{x}}}$. The original dimension of each vector $\mathbf{x}$ is 973 landmark genes. Additionally we use unpaired pre-treatment data (with no know post-treatment state) to improve learning of the latent representation. The graphical representation of PertVAE model is shown in Fig 1.1.

Joint distribution. PertVAE models joint $p(\mathbf{x}_1, \mathbf{x}_2, \mathbf{z}_1, \mathbf{z}_2)$, which is assumed to factorize as:

$$p(\mathbf{x}_1, \mathbf{x}_2, \mathbf{z}_1, \mathbf{z}_2) = p(\mathbf{x}_1|\mathbf{z}_1) \cdot p(\mathbf{x}_2|\mathbf{z}_2) \cdot p(\mathbf{z}_2|\mathbf{z}_1) \cdot p(\mathbf{z}_1) \tag{1.1}$$

Generative distribution p_θ. PertVAE's generative process is as follows:

$$p(\mathbf{z}_1) = \mathcal{N}(\mathbf{0}, \mathbf{I}) \tag{1.2}$$

$$p_{\theta_{\mathbf{z}_1\to\mathbf{z}_2}}(\mathbf{z}_2|\mathbf{z}_1) = \mathcal{N}\left(\mathbf{z}_2|\boldsymbol{\mu}_{\mathbf{z}_2} = f_\theta(\mathbf{z}_1), \boldsymbol{\sigma}^2_{\mathbf{z}_2} = \exp^{f_\theta(\mathbf{z}_1)}\right) \tag{1.3}$$

$$k \in \{1, 2\} : p_{\theta_{\mathbf{z}\to\mathbf{x}}}(\mathbf{x}_k|\mathbf{z}_k) = \mathcal{N}\left(\mathbf{x}_k|\boldsymbol{\mu}_{\mathbf{x}_k} = f_\theta(\mathbf{z}_k), \boldsymbol{\sigma}^2_{\mathbf{x}_k} = \exp^{f_\theta(\mathbf{z}_k)}\right) \tag{1.4}$$

The parameters of these distributions are computed by functions f_θ, which are neural networks with a total set of parameters θ. For brevity we refer to these parameters as θ instead of more specific subsets $\theta_{\mathbf{z}\to\mathbf{x}}$ or $\theta_{\mathbf{z}_1\to\mathbf{z}_2}$ when such level of detail unnecessarily clutters the notation.

We constrain the mean function in $p_{\theta_{\mathbf{z}_1\to\mathbf{z}_2}}$ to be a linear function $f_{\theta_{\mathbf{z}_1\to\mathbf{z}_2}}(\mathbf{z}_1)$ of the following form:

$$f_{\theta_{\mathbf{z}_1\to\mathbf{z}_2}}(\mathbf{z}_1) \equiv \mathbf{z}_1 + \mathbf{W}\mathbf{z}_1 + \mathbf{b} \tag{1.5}$$

with $\mathbf{W}$ and $\mathbf{b}$ initialized close to zero such that $f_{\theta_{\mathbf{z}_1\to\mathbf{z}_2}}(\mathbf{z}_1)$ starts as an identity function. We found that together with L2 penalization this formulation improves stability and generalization of the model.

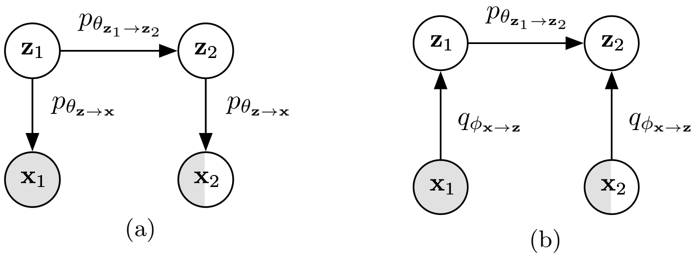

Figure 1.1: **Perturbation VAE.** (a) Factorization of the generative distribution p, (b) Factorization of the approximate posterior distribution q. Note, we use the generative $p_{\theta_{\mathbf{z}_1 \to \mathbf{z}_2}}$ in case $\mathbf{x}_2$ is not observed.

Approximate posterior q_ϕ. Depending on the type of the data, we assume the approximate posterior q with a set of parameters ϕ factorizes as:

$$\text{perturbation pairs:} \quad q_\phi(\mathbf{z}_1, \mathbf{z}_2 | \mathbf{x}_1, \mathbf{x}_2) = q_{\phi_{\mathbf{x} \to \mathbf{z}}}(\mathbf{z}_1 | \mathbf{x}_1) \cdot q_{\phi_{\mathbf{x} \to \mathbf{z}}}(\mathbf{z}_2 | \mathbf{x}_2) \tag{1.6}$$

$$\text{pre-treatment singleton:} \quad q_\phi(\mathbf{z}_1, \mathbf{z}_2, \mathbf{x}_2 | \mathbf{x}_1) = q_{\phi_{\mathbf{x} \to \mathbf{z}}}(\mathbf{z}_1 | \mathbf{x}_1) \cdot p_{\theta_{\mathbf{z}_1 \to \mathbf{z}_2}}(\mathbf{z}_2 | \mathbf{z}_1) \cdot p_{\theta_{\mathbf{z} \to \mathbf{x}}}(\mathbf{x}_2 | \mathbf{z}_2) \tag{1.7}$$

Analogously to the shared generative $p_{\theta_{\mathbf{z} \to \mathbf{x}}}$ distribution, also $q_{\phi_{\mathbf{x} \to \mathbf{z}}}(\mathbf{z}_k | \mathbf{x}_k)$ is shared for both $k \in \{1, 2\}$ and takes from of a diagonal Gaussian:

$$k \in \{1, 2\} : q_{\phi_{\mathbf{x} \to \mathbf{z}}}(\mathbf{z}_k | \mathbf{x}_k) = \mathcal{N}\left(\mathbf{z}_k | \boldsymbol{\mu}_{\mathbf{z}_k} = f_\phi(\mathbf{x}_k), \boldsymbol{\sigma}^2_{\mathbf{z}_k} = \exp^{f_\phi(\mathbf{x}_k)}\right) \tag{1.8}$$

Fitting θ and ϕ parameters. We jointly optimize the generative model θ and variational ϕ parameters to maximize evidence lower bound, $\text{ELBO}_{\text{PertVAE}}$, of training data. The training data consists of a set of perturbation pairs P and unpaired "singleton" examples S that we leverage to train the latent space variational autoencoder as well.

$$\sum_{(\mathbf{x}_1, \mathbf{x}_2) \in P} \log p\left(\mathbf{x}_1, \mathbf{x}_2\right) + \sum_{\mathbf{x}_1 \in S} \log p\left(\mathbf{x}_1\right) \geq \text{ELBO}_{\text{PertVAE}} \tag{1.9}$$

$$\text{ELBO}_{\text{PertVAE}} = \sum_{(\mathbf{x}_1, \mathbf{x}_2) \in P} \mathcal{L}_P\left(\mathbf{x}_1, \mathbf{x}_2; \theta, \phi\right) + \sum_{\mathbf{x}_1 \in S} \mathcal{L}_S\left(\mathbf{x}_1; \theta, \phi\right) \tag{1.10}$$

The individual per-example lower bounds $\mathcal{L}_P$ and $\mathcal{L}_S$ take the following form:

$$\begin{aligned}
\mathcal{L}_P(\mathbf{x}_1, \mathbf{x}_2;\ \theta, \phi) &= \mathbb{E}_{q_\phi(\mathbf{z}_1, \mathbf{z}_2 | \mathbf{x}_1, \mathbf{x}_2)}\big[\log p_\theta(\mathbf{x}_1, \mathbf{x}_2, \mathbf{z}_1, \mathbf{z}_2) - \log q_\phi(\mathbf{z}_1, \mathbf{z}_2 | \mathbf{x}_1, \mathbf{x}_2)\big] && (1.11)\\
&= \mathbb{E}_{q_\phi(\mathbf{z}_1 | \mathbf{x}_1)}\big[\log p_\theta(\mathbf{x}_1 | \mathbf{z}_1) - D_{KL}\left[q_\phi(\mathbf{z}_2 | \mathbf{x}_2) || p_\theta(\mathbf{z}_2 | \mathbf{z}_1)\right]\big] && (1.12)\\
&\quad + \mathbb{E}_{q_\phi(\mathbf{z}_2 | \mathbf{x}_2)}\left[\log p_\theta(\mathbf{x}_2 | \mathbf{z}_2)\right]\\
&\quad - D_{KL}\left[q_\phi(\mathbf{z}_1 | \mathbf{x}_1) || p(\mathbf{z}_1)\right]\\
\mathcal{L}_S(\mathbf{x}_1;\ \theta, \phi) &= \mathbb{E}_{q_\phi(\mathbf{z}_1 | \mathbf{x}_1)}\big[\log p_\theta(\mathbf{x}_1, \mathbf{z}_1) - \log q_\phi(\mathbf{z}_1 | \mathbf{x}_1)\big] && (1.13)\\
&= \mathbb{E}_{q_\phi(\mathbf{z}_1 | \mathbf{x}_1)}\left[\log p_\theta(\mathbf{x}_1 | \mathbf{z}_1)\right] - D_{KL}\left[q_\phi(\mathbf{z}_1 | \mathbf{x}_1) || p(\mathbf{z}_1)\right]
\end{aligned}$$

The expectations that are part of our evidence lower bounds are evaluated approximately, by Monte Carlo sampling. In practice we use two MC samples. Thanks to so-called reparametrization trick it

is possible to backpropagate through such approximation of the expectations, yielding an unbiased gradient estimator of the distribution parameters known as Stochastic Gradient Variational Bayes (SGVB) [1]. In our case, no approximation is necessary for evaluation of the Kullback–Leibler divergences present in the ELBO, as there is a close form solution for D_{KL} between two normal distributions. Now we have all the tools to evaluate $\mathrm{ELBO}_{\mathrm{PertVAE}}$ and compute its approximate gradients w.r.t. both θ and ϕ parameters. We then use Adam [2] to compute the parameter updates during the optimization process.

1.2 Drug response variational autoencoder

Analogously to semi-supervised variational autoencoder, we extended the unsupervised Perturbation VAE to a semi-supervised model by incorporating a modified "M2 model" [3]. The extended model, drug response variational autoencoder (Dr.VAE), enables us to model both drug-induced perturbation effects as well as treatment response outcome at the same time. We train Dr.VAE jointly and not as a stack of two models PertVAE + M2 model, similarly to how semi-supervised VAE can be trained jointly [4].

We use similar type of data to train Dr.VAE as we use for PertVAE, however some of the perturbation pairs and pre-treatment singletons now can have a binary outcome label $\mathbf{y}$ associated with them, denoting if the drug treatment was successful or not. Schema of Dr.VAE model is shown in main text, Fig 1.

Joint distribution. Drug response VAE extends PertVAE to model a joint distribution $p(\mathbf{x}_1, \mathbf{x}_2, \mathbf{z}_1, \mathbf{z}_2, \mathbf{z}_3, \mathbf{y})$ factorized as:

$$p(\mathbf{x}_1, \mathbf{x}_2, \mathbf{z}_1, \mathbf{z}_2, \mathbf{z}_3, \mathbf{y}) = p(\mathbf{x}_1|\mathbf{z}_1) \cdot p(\mathbf{x}_2|\mathbf{z}_2) \cdot p(\mathbf{z}_2|\mathbf{z}_1) \cdot p(\mathbf{z}_1|\mathbf{z}_3, \mathbf{y}) \cdot p(\mathbf{z}_3) \cdot p(\mathbf{y}) \tag{1.14}$$

Generative distributions p_θ. The individual generative distributions, Dr.VAE factorizes to, have the following form:

$$p(\mathbf{y}) = \mathrm{Cat}(\mathbf{y}|\boldsymbol{\pi} = \mathrm{Unif}) \tag{1.15}$$

$$p(\mathbf{z}_3) = \mathcal{N}(\mathbf{0}, \mathbf{I}) \tag{1.16}$$

$$p_\theta(\mathbf{z}_1|\mathbf{z}_3, \mathbf{y}) = \mathcal{N}\left(\mathbf{z}_1|\boldsymbol{\mu}_{\mathbf{z}_1} = f_\theta(\mathbf{z}_3, \mathbf{y}), \boldsymbol{\sigma}^2_{\mathbf{z}_1} = \exp^{f_\theta(\mathbf{z}_3, \mathbf{y})}\right) \tag{1.17}$$

$$p_\theta(\mathbf{z}_2|\mathbf{z}_1) = \mathcal{N}\left(\mathbf{z}_2|\boldsymbol{\mu}_{\mathbf{z}_2} = f_\theta(\mathbf{z}_1), \boldsymbol{\sigma}^2_{\mathbf{z}_2} = \exp^{f_\theta(\mathbf{z}_1)}\right) \tag{1.18}$$

$$k \in \{1, 2\} : p_\theta(\mathbf{x}_k|\mathbf{z}_k) = \mathcal{N}\left(\mathbf{x}_k|\boldsymbol{\mu}_{\mathbf{x}_k} = f_\theta(\mathbf{z}_k), \boldsymbol{\sigma}^2_{\mathbf{x}_k} = \exp^{f_\theta(\mathbf{z}_k)}\right) \tag{1.19}$$

Same way as in PertVAE, we share the "data decoder" $p_\theta(\mathbf{x}_k|\mathbf{z}_k)$ among both $k \in \{1, 2\}$.

Approximate posterior q_ϕ. Depending on the type of the data, we assume the approximate posterior q to factorize as:

$$\text{labeled pair: } q_\phi(\mathbf{z}_1,\mathbf{z}_2,\mathbf{z}_3|\mathbf{x}_1,\mathbf{x}_2,\mathbf{y}) = q_\phi(\mathbf{z}_1|\mathbf{x}_1)\cdot q_\phi(\mathbf{z}_2|\mathbf{x}_2)\cdot q_\phi(\mathbf{z}_3|\mathbf{z}_1,\mathbf{y}) \quad (1.20)$$

$$\text{unlabeled pair: } q_\phi(\mathbf{z}_1,\mathbf{z}_2,\mathbf{z}_3,\mathbf{y}|\mathbf{x}_1,\mathbf{x}_2) = q_\phi(\mathbf{z}_1|\mathbf{x}_1)\cdot q_\phi(\mathbf{z}_2|\mathbf{x}_2)\cdot q_\phi(\mathbf{y}|\mathbf{z}_1,\mathbf{z}_2)\cdot q_\phi(\mathbf{z}_3|\mathbf{z}_1,\mathbf{y}) \quad (1.21)$$

$$\text{labeled singleton: } q_\phi(\mathbf{z}_1,\mathbf{z}_2,\mathbf{z}_3,\mathbf{x}_2|\mathbf{x}_1,\mathbf{y}) = q_\phi(\mathbf{z}_1|\mathbf{x}_1)\cdot p_\theta(\mathbf{z}_2|\mathbf{z}_1)\cdot p_\theta(\mathbf{x}_2|\mathbf{z}_2)\cdot q_\phi(\mathbf{z}_3|\mathbf{z}_1,\mathbf{y}) \quad (1.22)$$

$$\text{unlab. singleton: } q_\phi(\mathbf{z}_1,\mathbf{z}_2,\mathbf{z}_3,\mathbf{x}_2,\mathbf{y}|\mathbf{x}_1) = q_\phi(\mathbf{z}_1|\mathbf{x}_1)\cdot p_\theta(\mathbf{z}_2|\mathbf{z}_1)\cdot p_\theta(\mathbf{x}_2|\mathbf{z}_2)\cdot \quad (1.23)$$
$$\cdot\, q_\phi(\mathbf{y}|\mathbf{z}_1,\mathbf{z}_2)\cdot q_\phi(\mathbf{z}_3|\mathbf{z}_1,\mathbf{y})$$

The "data encoder" $k \in \{1,2\} : q_\phi(\mathbf{z}_k|\mathbf{x}_k)$ is shared and parametrized the same way as in PertVAE. The additional approximate posterior distributions then take the following form:

$$q_\phi(\mathbf{y}|\mathbf{z}_1,\mathbf{z}_2) = \mathrm{Cat}\big(\mathbf{y}|\boldsymbol{\pi} = \mathrm{softmax}(f_\phi(\mathbf{z}_1,\mathbf{z}_2-\mathbf{z}_1))\big) \quad (1.24)$$

$$q_\phi(\mathbf{z}_3|\mathbf{z}_1,\mathbf{y}) = \mathcal{N}\left(\mathbf{z}_3|\boldsymbol{\mu}_{\mathbf{z}_3} = f_\phi(\mathbf{z}_1,\mathbf{y}), \boldsymbol{\sigma}^2_{\mathbf{z}_3} = \exp^{f_\phi(\mathbf{z}_1,\mathbf{y})}\right) \quad (1.25)$$

The afford mentioned factorizations of the joint and of the posteriors also provide a recipe for sampling and inference in the model by Monte Carlo sampling.

Fitting θ and ϕ parameters. We have 4 different sets of partially observed variables, which correspond to different types of data. Therefore there are 4 different evidence lower bounds to optimize:

$$\text{labeled perturbation pairs } LP\text{:} \quad \sum \mathcal{L}_{LP}(\mathbf{x}_1,\mathbf{x}_2,\mathbf{y};\theta,\phi) \quad (1.26)$$

$$\text{unlabeled perturbation pairs } UP\text{:} \quad \sum \mathcal{L}_{UP}(\mathbf{x}_1,\mathbf{x}_2;\theta,\phi) \quad (1.27)$$

$$\text{labeled pre-treatment singletons } LS\text{:} \quad \sum \mathcal{L}_{LS}(\mathbf{x}_1,\mathbf{y};\theta,\phi) \quad (1.28)$$

$$\text{unlabeled pre-treatment singletons } US\text{:} \quad \sum \mathcal{L}_{US}(\mathbf{x}_1;\theta,\phi) \quad (1.29)$$

The sum of these 4 specific evidence lower bounds, $\mathrm{ELBO}_{\mathrm{DrVAE}}$, is the evidence lower bound we need to maximize. The derivation of these specific lower bounds follows the same principles as shown above for PertVAE and as shown in the derivation of semi-supervised VAE [3, 4]. Particularly, for unlabeled and labeled perturbation pairs, denoted UP and LP respectively, we obtain the following

bounds:

$$\begin{aligned}
\mathcal{L}_{UP}(\mathbf{x}_1, \mathbf{x}_2;\ \theta, \phi) &= \mathbb{E}_{q_\phi(\mathbf{z}_1,\mathbf{z}_2,\mathbf{z}_3,\mathbf{y}|\mathbf{x}_1,\mathbf{x}_2)}\big[\log p_\theta(\mathbf{x}_1, \mathbf{x}_2, \mathbf{z}_1, \mathbf{z}_2, \mathbf{z}_3, \mathbf{y}) - \\
&\qquad - \log q_\phi(\mathbf{z}_1, \mathbf{z}_2, \mathbf{z}_3, \mathbf{y}|\mathbf{x}_1, \mathbf{x}_2)\big] && (1.30)\\
&= \mathbb{E}_{q_\phi(\mathbf{z}_1|\mathbf{x}_1)}\big[\log p_\theta(\mathbf{x}_1|\mathbf{z}_1) - D_{KL}\left[q_\phi(\mathbf{z}_2|\mathbf{x}_2)||p_\theta(\mathbf{z}_2|\mathbf{z}_1)\right]\big] && (1.31)\\
&\quad + \mathbb{E}_{q_\phi(\mathbf{z}_2|\mathbf{x}_2)}\left[\log p_\theta(\mathbf{x}_2|\mathbf{z}_2)\right]\\
&\quad + \mathbb{E}_{q_\phi(\mathbf{y}|\mathbf{z}_1,\mathbf{z}_2)q_\phi(\mathbf{z}_2|\mathbf{x}_2)q_\phi(\mathbf{z}_3|\mathbf{z}_1,\mathbf{y})}\big[-D_{KL}\left[q_\phi(\mathbf{z}_1|\mathbf{x}_1)||p_\theta(\mathbf{z}_1|\mathbf{z}_3, \mathbf{y})\right]\big]\\
&\quad + \mathbb{E}_{q_\phi(\mathbf{y}|\mathbf{z}_1,\mathbf{z}_2)q_\phi(\mathbf{z}_1|\mathbf{x}_1)q_\phi(\mathbf{z}_2|\mathbf{x}_2)}\big[-D_{KL}\left[q_\phi(\mathbf{z}_3|\mathbf{z}_1, \mathbf{y})||p(\mathbf{z}_3)\right]\big]\\
&\quad + \mathbb{E}_{q_\phi(\mathbf{z}_1|\mathbf{x}_1)q_\phi(\mathbf{z}_2|\mathbf{x}_2)}\big[-D_{KL}\left[q_\phi(\mathbf{y}|\mathbf{z}_1, \mathbf{z}_2)||p(\mathbf{y})\right]\big]\\
\mathcal{L}_{LP}(\mathbf{x}_1, \mathbf{x}_2, \mathbf{y};\ \theta, \phi) &= \mathbb{E}_{q_\phi(\mathbf{z}_1,\mathbf{z}_2,\mathbf{z}_3|\mathbf{x}_1,\mathbf{x}_2,\mathbf{y})}\big[\log p_\theta(\mathbf{x}_1, \mathbf{x}_2, \mathbf{z}_1, \mathbf{z}_2, \mathbf{z}_3, \mathbf{y}) - \\
&\qquad - \log q_\phi(\mathbf{z}_1, \mathbf{z}_2, \mathbf{z}_3|\mathbf{x}_1, \mathbf{x}_2, \mathbf{y})\big] && (1.32)\\
&= \mathbb{E}_{q_\phi(\mathbf{z}_1|\mathbf{x}_1)}\big[\log p_\theta(\mathbf{x}_1|\mathbf{z}_1) - D_{KL}\left[q_\phi(\mathbf{z}_2|\mathbf{x}_2)||p_\theta(\mathbf{z}_2|\mathbf{z}_1)\right]\big] && (1.33)\\
&\quad + \mathbb{E}_{q_\phi(\mathbf{z}_2|\mathbf{x}_2)}\left[\log p_\theta(\mathbf{x}_2|\mathbf{z}_2)\right]\\
&\quad + \mathbb{E}_{q_\phi(\mathbf{z}_3|\mathbf{z}_1,\mathbf{y})}\big[-D_{KL}\left[q_\phi(\mathbf{z}_1|\mathbf{x}_1)||p_\theta(\mathbf{z}_1|\mathbf{z}_3, \mathbf{y})\right]\big]\\
&\quad + \mathbb{E}_{q_\phi(\mathbf{z}_1|\mathbf{x}_1)}\big[-D_{KL}\left[q_\phi(\mathbf{z}_3|\mathbf{z}_1, \mathbf{y})||p(\mathbf{z}_3)\right]\big]\\
&\quad + \log p(\mathbf{y})
\end{aligned}$$

Evidence lower bounds for unlabeled singletons (US) and labeled singletons (LS):

$$\begin{aligned}
\mathcal{L}_{US}(\mathbf{x}_1;\ \theta, \phi) &= \mathbb{E}_{q_\phi(\mathbf{z}_1,\mathbf{z}_2,\mathbf{z}_3,\mathbf{y}|\mathbf{x}_1)}\big[\log p_\theta(\mathbf{x}_1, \mathbf{z}_1, \mathbf{z}_2, \mathbf{z}_3, \mathbf{y}) - \\
&\qquad - \log q_\phi(\mathbf{z}_1, \mathbf{z}_2, \mathbf{z}_3, \mathbf{y}|\mathbf{x}_1)\big] && (1.34)\\
&= \mathbb{E}_{q_\phi(\mathbf{z}_1|\mathbf{x}_1)}\left[\log p_\theta(\mathbf{x}_1|\mathbf{z}_1)\right] && (1.35)\\
&\quad + \mathbb{E}_{q_\phi(\mathbf{y}|\mathbf{z}_1,\mathbf{z}_2)p_\theta(\mathbf{z}_2|\mathbf{z}_1)q_\phi(\mathbf{z}_1|\mathbf{x}_1)q_\phi(\mathbf{z}_3|\mathbf{z}_1,\mathbf{y})}\big[-D_{KL}\left[q_\phi(\mathbf{z}_1|\mathbf{x}_1)||p_\theta(\mathbf{z}_1|\mathbf{z}_3, \mathbf{y})\right]\big]\\
&\quad + \mathbb{E}_{q_\phi(\mathbf{y}|\mathbf{z}_1,\mathbf{z}_2)q_\phi(\mathbf{z}_1|\mathbf{x}_1)p_\theta(\mathbf{z}_2|\mathbf{z}_1)}\big[-D_{KL}\left[q_\phi(\mathbf{z}_3|\mathbf{z}_1, \mathbf{y})||p(\mathbf{z}_3)\right]\big]\\
&\quad + \mathbb{E}_{q_\phi(\mathbf{z}_1|\mathbf{x}_1)p_\theta(\mathbf{z}_2|\mathbf{z}_1)}\big[-D_{KL}\left[q_\phi(\mathbf{y}|\mathbf{z}_1, \mathbf{z}_2)||p(\mathbf{y})\right]\big]\\
\mathcal{L}_{LS}(\mathbf{x}_1, \mathbf{y};\ \theta, \phi) &= \mathbb{E}_{q_\phi(\mathbf{z}_1,\mathbf{z}_2,\mathbf{z}_3|\mathbf{x}_1,\mathbf{y})}\big[\log p_\theta(\mathbf{x}_1, \mathbf{z}_1, \mathbf{z}_2, \mathbf{z}_3, \mathbf{y}) - \\
&\qquad - \log q_\phi(\mathbf{z}_1, \mathbf{z}_2, \mathbf{z}_3|\mathbf{x}_1, \mathbf{y})\big] && (1.36)\\
&= \mathbb{E}_{q_\phi(\mathbf{z}_1|\mathbf{x}_1)}\left[\log p_\theta(\mathbf{x}_1|\mathbf{z}_1)\right] && (1.37)\\
&\quad + \mathbb{E}_{q_\phi(\mathbf{z}_3|\mathbf{z}_1,\mathbf{y})q_\phi(\mathbf{z}_1|\mathbf{x}_1)}\big[-D_{KL}\left[q_\phi(\mathbf{z}_1|\mathbf{x}_1)||p_\theta(\mathbf{z}_1|\mathbf{z}_3, \mathbf{y})\right]\big]\\
&\quad + \mathbb{E}_{q_\phi(\mathbf{z}_1|\mathbf{x}_1)}\big[-D_{KL}\left[q_\phi(\mathbf{z}_3|\mathbf{z}_1, \mathbf{y})||p(\mathbf{z}_3)\right]\big]\\
&\quad + \log p(\mathbf{y})
\end{aligned}$$

In these lower bounds, we compute expectation w.r.t. $q_\phi(\mathbf{y}|\mathbf{z}_1, \mathbf{z}_2)$ exactly by summation as $\mathbf{y}$ is in our case a binary random variable:

$$\mathbb{E}_{q_\phi(\mathbf{y}|\mathbf{z}_1,\mathbf{z}_2)}[f(\mathbf{y}, \cdot)] = \sum_{t\in\{0,1\}} q_\phi(\mathbf{y} = t|\mathbf{z}_1, \mathbf{z}_2) f(\mathbf{y}, \cdot) \tag{1.38}$$

Note, that the Kullback–Leibler divergence of two categorical distributions over $\mathbf{y}$ can be computed analytically. Moreover, to mitigate the problem of overly strong prior causing the optimization to

get stuck in bad local optima, we follow [5] and allow "free bits" in D_{KL} of approximate posterior and prior over $\mathbf{y}$ and $\mathbf{z}_3$ variables. Now, using the approach as described previously for PertVAE, we can evaluate $\text{ELBO}_{\text{DrVAE}}$ and its gradients w.r.t. both θ and ϕ parameters.

Next, akin to semi-supervised VAE [3], we need to explicitly introduce loss of the predictive posterior $\log q_\phi(\mathbf{y}|\mathbf{z}_1, \mathbf{z}_2)$ in order for it to be trained on labeled data as well. This is required since in the labeled data the random variable $\mathbf{y}$ is an observed variable and therefore the lower bounds $\mathcal{L}_{LP}$ and $\mathcal{L}_{LS}$ are conditioned on $\mathbf{y}$ and do not contribute to training of $q_\phi(\mathbf{y}|\mathbf{z}_1, \mathbf{z}_2)$.

Additionally, we found beneficial to include explicit perturbation prediction loss $\mathbb{E}_{q_\phi(\mathbf{z}_1|\mathbf{x}_1)p_\theta(\mathbf{z}_2|\mathbf{z}_1)}\left[\log p_\theta(\mathbf{x}_2|\mathbf{z}_2)\right]$ in addition to minimization of KL divergence between the approximate posterior and predicted distribution over $\mathbf{z}_2$, $\mathbb{E}_{q_\phi(\mathbf{z}_1|\mathbf{x}_1)}\left[D_{KL}\left[q_\phi(\mathbf{z}_2|\mathbf{x}_2)||p_\theta(\mathbf{z}_2|\mathbf{z}_1)\right]\right]$, that is a part of $\mathcal{L}_{UP}$ and $\mathcal{L}_{LP}$.

Eventually, the final objective $\mathcal{J}_{\text{DrVAE}}$ we seek to maximize is

$$\begin{aligned}\mathcal{J}_{\text{DrVAE}} = \text{ELBO}_{\text{DrVAE}} &+ \\ &+ \alpha \sum_{LP \cup LS} \mathbb{E}_{q_\phi(\mathbf{z}_1|\mathbf{x}_1)p_\theta(\mathbf{z}_2|\mathbf{z}_1)}\left[\log q_\phi(\mathbf{y}=\mathbf{t}|\mathbf{z}_1, \mathbf{z}_2)\right] + \\ &+ \beta \sum_{LP \cup UP} \mathbb{E}_{q_\phi(\mathbf{z}_1|\mathbf{x}_1)p_\theta(\mathbf{z}_2|\mathbf{z}_1)}\left[\log p_\theta(\mathbf{x}_2|\mathbf{z}_2)\right]\end{aligned} \tag{1.39}$$

where α and β are hyperparameters weighting the classification and perturbation loss, respectively, relative to the $\text{ELBO}_{\text{DrVAE}}$ and are selected based on the classification performance on a validation set distinct from a test set. We found $\beta = 0.05$ to be well suited for all drugs, while α is found for each drug individually by cross-validation in grid search over $\{1, 3, 5, 10\}$.

Bibliography

[1] Kingma DP, Welling M. Auto-encoding variational bayes. arXiv preprint arXiv:13126114. 2013;.

[2] Kingma D, Ba J. Adam: A method for stochastic optimization. arXiv preprint arXiv:14126980. 2014;.

[3] Kingma DP, Mohamed S, Rezende DJ, Welling M. Semi-supervised learning with deep generative models. In: Advances in Neural Information Processing Systems; 2014. p. 3581–3589.

[4] Louizos C, Swersky K, Li Y, Welling M, Zemel R. The variational fair autoencoder. arXiv preprint arXiv:151100830. 2015;.

[5] Kingma DP, Salimans T, Welling M. Improving variational inference with inverse autoregressive flow. arXiv preprint arXiv:160604934. 2016;.

Supplementary Results

2.1 Effect-to-replicate variance ratio in perturbation experiments

The tested set of drugs manifests considerable diversity in the type of drugs (cytotoxic or targeted) and number of available perturbation experiments ranging from 32 to 417 in as few as 8 and up to 60 distinct cell lines. The magnitude of the perturbation effect together with the number of available experiments and variance in biological replicates have paramount impact on how well our Dr.VAE can model these drug perturbations. To quantify this connection, we first need to quantify the drug perturbation effect.

We computed the effect-to-replicate variance ratio (ERVR) for perturbation experiments of each cell line with at least two biological replicates as the ratio of variance between clusters to the variance within clusters where we denote biological replicates of the control (pre-treatment) gene expression as one cluster and the matched post-treatment gene expression replicates as the second cluster. For a drug d with perturbation experiments on a set of cell lines C_d its effect-to-replicate variance ratio is

$$\mathrm{ERVR}_d = \frac{1}{|C_d|}\sum_{c\in C_d} \frac{Var \text{ between } S_c^{pre} \text{ and } S_c^{post}}{Var(S_c^{pre})+Var(S_c^{post})} \tag{2.1}$$

$$= \frac{1}{|C_d|}\sum_{c\in C_d} \frac{Var(S_c)-Var(S_c^{pre})-Var(S_c^{post})}{Var(S_c^{pre})+Var(S_c^{post})} \tag{2.2}$$

where $S_c = S_c^{pre} \cup S_c^{post}$ is the set of gene expression measurements of cell line c composed of replicates of its pre- and post-treatment experiments for drug d, S_c^{pre} and S_c^{post}, respectively.

The computed ERVR for each drug is listed in Supplementary Table S6. We analyzed the impact of ERVR in the results section of the main paper.

2.2 Reconstruction of gene expression from latent representation

Dr.VAE learns a non-linear representation of expression of the 1000 landmark genes in a reduced 100 dimensional latent space. In the above we studied how this embedding fairs in drug response classification and drug perturbation prediction. Lastly, we evaluated how well Dr.VAE can reconstruct the original gene expression from this reduced representation for a held out set of cell lines. We computed RMSE and Pearson correlation of the reconstructed gene expression on the test set of CTRPv2 cell lines and compared it to PCA reconstruction from the first 100 principal components. The data splitting and training of Dr.VAE and PCA were performed the same way as in the other experiments described in the main paper.

Dr.VAE accomplished average gene expression reconstruction RMSE of 0.380 per gene, while PCA managed 0.329. In terms of Pearson correlation, the reconstructed gene expression from Dr.VAE and PCA correlated with the original expression levels with ρ equal 0.767 and 0.829, respectively. While Dr.VAE was trained for three tasks concurrently with model selection focused on the classification task, it still achieved good reconstruction accuracy. This shows that Dr.VAE does indeed learn latent representation of gene expression.

Supplementary Figures and Tables

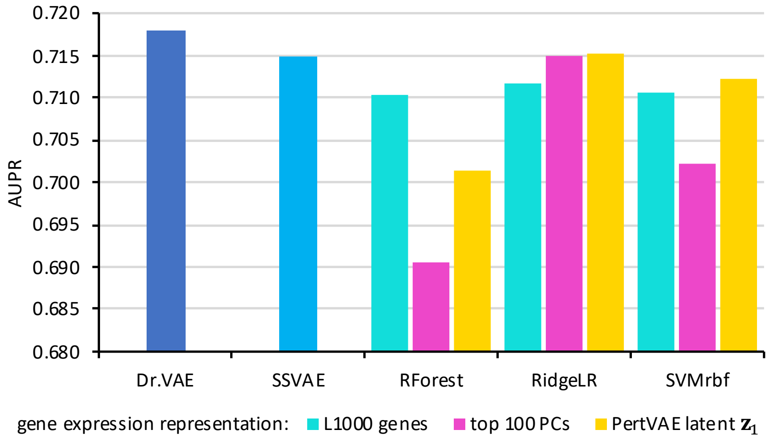

(a)

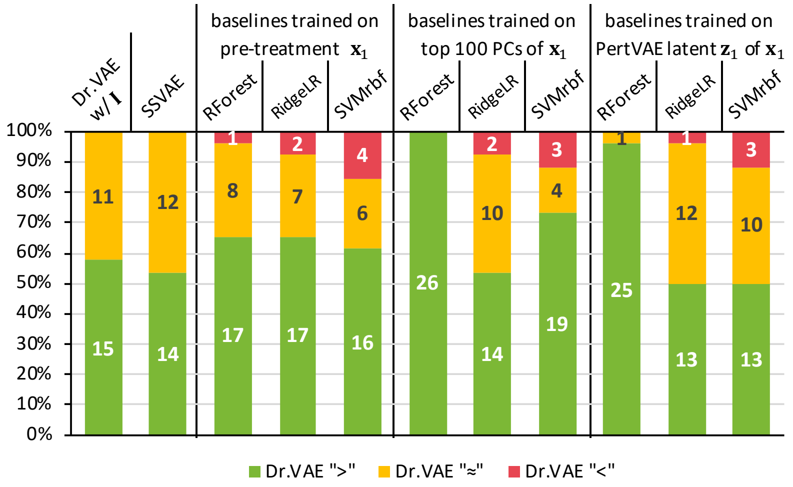

(b)

Figure S1: **Summarized classification results (AUPR).** (a) Area under the precision-recall curve of Dr.VAE and baseline methods. Shown is average over 26 drugs, each evaluated in 100 train-validation-test splits. (b) Dr.VAE is comparable or better than any other baseline for >85% of the drugs (p-val < 0.05 Wilcoxon test).

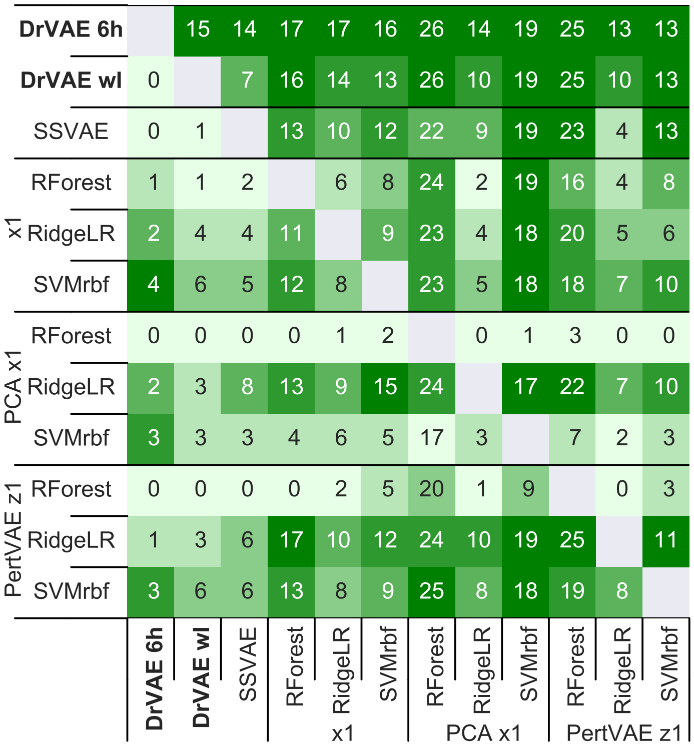

Figure S2: **All to all comparison of tested methods (AUPR).** Comparison of all tested methods by one-sided Wilcoxon Signed-Rank Test (p-val < 0.05) based on test area under the precision-recall curve performance in 100 train-validation-test splits. Cell at (i, j) position shows the number of drugs for which a method in row i outperforms the method corresponding to j-th column. Analogous to Fig 4 in the main text that presents comparison by test AUROC.

Table S1: **Per-drug AUROC classification results.** Cross-validated test AUROC (area under the ROC curve) of our Dr.VAE to SSVAE and other classification models detailed for each evaluated drug. Methods including PCA and PertVAE are 2-step methods: (i) fit the unsupervised model, (ii) use latent representation to fit a standard classifier.

**AUROC**				baselines trained on pre-treatment $\mathbf{x}_1$			baselines trained on top 100 PCs of $\mathbf{x}_1$			baselines trained on PertVAE latent $\mathbf{z}_1$ of $\mathbf{x}_1$		
drug	**Dr.VAE (6h)**	**Dr.VAE w/ I**	SSVAE	RForest	RidgeLR	SVMrbf	RForest	RidgeLR	SVMrbf	RForest	RidgeLR	SVMrbf
bortezomib	0.670	0.669	0.663	0.659	0.649	0.641	0.629	0.648	0.644	0.649	0.666	0.669
bosutinib	0.743	0.743	0.736	0.737	0.750	0.735	0.714	0.758	0.749	0.729	0.747	0.735
ciclosporin	0.638	0.637	0.642	0.632	0.638	0.637	0.622	0.636	0.640	0.619	0.631	0.633
clofarabine	0.757	0.755	0.754	0.746	0.745	0.749	0.723	0.746	0.738	0.742	0.754	0.754
dasatinib	0.775	0.774	0.773	0.764	0.771	0.778	0.768	0.776	0.784	0.756	0.772	0.775
decitabine	0.840	0.838	0.838	0.827	0.832	0.822	0.803	0.838	0.810	0.818	0.839	0.820
docetaxel	0.733	0.729	0.724	0.735	0.719	0.711	0.669	0.725	0.696	0.712	0.725	0.724
etoposide	0.866	0.863	0.868	0.847	0.869	0.874	0.832	0.867	0.843	0.846	0.864	0.866
fluvastatin	0.650	0.648	0.649	0.657	0.660	0.672	0.643	0.654	0.651	0.642	0.647	0.652
fulvestrant	0.561	0.559	0.549	0.578	0.551	0.549	0.550	0.554	0.552	0.551	0.545	0.547
gemcitabine	0.734	0.734	0.728	0.726	0.721	0.732	0.715	0.734	0.721	0.723	0.726	0.729
lovastatin	0.651	0.651	0.645	0.647	0.646	0.650	0.619	0.645	0.631	0.639	0.655	0.651
mitomycin	0.734	0.731	0.732	0.729	0.694	0.733	0.716	0.723	0.728	0.722	0.715	0.738
niclosamide	0.687	0.688	0.683	0.678	0.670	0.669	0.659	0.675	0.677	0.666	0.669	0.685
omacetaxine me	0.735	0.730	0.725	0.709	0.729	0.726	0.673	0.732	0.718	0.708	0.737	0.738
paclitaxel	0.753	0.751	0.749	0.744	0.729	0.735	0.723	0.739	0.732	0.739	0.746	0.753
PLX-4032	0.583	0.583	0.585	0.577	0.582	0.559	0.570	0.582	0.579	0.574	0.591	0.592
prochlorperazine	0.609	0.608	0.603	0.593	0.610	0.609	0.577	0.614	0.603	0.595	0.605	0.594
sirolimus	0.670	0.670	0.672	0.668	0.645	0.654	0.652	0.656	0.665	0.665	0.670	0.683
sitagliptin	0.582	0.581	0.586	0.556	0.561	0.564	0.554	0.566	0.572	0.565	0.569	0.594
teniposide	0.739	0.737	0.737	0.744	0.729	0.737	0.708	0.732	0.710	0.725	0.726	0.725
topotecan	0.752	0.748	0.752	0.738	0.743	0.748	0.726	0.742	0.734	0.737	0.746	0.743
trifluoperazine	0.659	0.657	0.651	0.676	0.658	0.648	0.635	0.661	0.647	0.640	0.666	0.647
valdecoxib	0.708	0.707	0.704	0.703	0.708	0.674	0.689	0.707	0.689	0.690	0.706	0.688
vincristine	0.788	0.786	0.792	0.777	0.773	0.786	0.765	0.779	0.777	0.778	0.786	0.794
vorinostat	0.743	0.742	0.734	0.732	0.738	0.731	0.714	0.741	0.725	0.724	0.739	0.726
MEAN	0.706	0.704	0.703	0.699	0.697	0.697	0.679	0.701	0.693	0.691	0.702	0.702

Table S2: **Per-drug statistical comparison of Dr.VAE to other evaluated methods by AUROC**. Statistical comparison of Dr.VAE to a set of evaluated baseline models on basis of their test AUROC on 100 data splits. Shown is p-value of one-sided Wilcoxon Signed-Rank Test rejecting null hypothesis of "Dr.VAE performance is worse or no different from the compared model performance" in favor of alternative hypothesis "Dr.VAE mean performance is higher than the compared baseline model". In Dr.VAE column, the mean test AUROC is shown.

**AUROC**				baselines trained on pre-treatment $\mathbf{x}_1$			baselines trained on top 100 PCs of $\mathbf{x}_1$			baselines trained on PertVAE latent $\mathbf{z}_1$ of $\mathbf{x}_1$		
drug	**Dr.VAE (6h)**	**Dr.VAE w/ I**	SSVAE	RForest	RidgeLR	SVMrbf	RForest	RidgeLR	SVMrbf	RForest	RidgeLR	SVMrbf
bortezomib	0.670	6.2E-03	1.9E-03	1.3E-03	4.5E-09	4.3E-12	5.5E-13	1.7E-10	2.9E-10	5.1E-07	1.5E-01	4.3E-01
bosutinib	0.743	1.2E-02	3.4E-04	3.4E-02	1.0E+00	4.2E-03	8.4E-11	1.0E+00	9.4E-01	2.0E-05	9.0E-01	2.7E-03
ciclosporin	0.638	1.5E-01	8.9E-01	1.2E-01	4.5E-01	4.8E-01	4.9E-04	3.8E-01	8.5E-01	4.2E-04	9.1E-02	2.5E-01
clofarabine	0.757	2.1E-06	8.8E-02	2.9E-05	6.1E-04	4.8E-05	1.7E-15	2.7E-06	3.0E-11	2.5E-08	1.9E-01	6.3E-02
dasatinib	0.775	1.9E-04	9.4E-02	1.9E-06	5.4E-02	9.7E-01	1.3E-03	7.2E-01	1.0E+00	4.9E-11	1.3E-02	1.4E-01
decitabine	0.840	6.0E-07	2.8E-01	1.0E-07	6.9E-05	2.6E-11	3.1E-15	4.9E-02	4.9E-16	1.6E-12	3.6E-01	2.9E-14
docetaxel	0.733	8.5E-06	2.1E-03	5.2E-01	2.0E-03	9.2E-08	3.3E-15	2.5E-02	1.3E-11	1.4E-04	2.1E-02	7.7E-03
etoposide	0.866	6.1E-09	7.7E-01	3.6E-12	9.9E-01	1.0E+00	1.9E-16	6.1E-01	4.1E-14	5.8E-13	1.2E-01	4.5E-01
fluvastatin	0.650	7.7E-09	3.6E-01	9.8E-01	1.0E+00	1.0E+00	2.1E-02	8.5E-01	5.3E-01	3.2E-03	7.1E-02	7.1E-01
fulvestrant	0.561	1.3E-01	9.4E-02	9.8E-01	5.8E-02	8.4E-02	1.7E-01	1.7E-01	2.1E-01	8.9E-02	3.9E-02	4.1E-02
gemcitabine	0.734	4.1E-01	5.4E-03	8.0E-03	9.5E-04	3.0E-01	5.1E-07	6.8E-01	2.0E-06	1.2E-04	1.1E-03	1.2E-02
lovastatin	0.651	3.4E-01	6.4E-02	1.1E-01	1.3E-02	2.6E-01	3.4E-12	1.8E-03	3.0E-09	1.2E-03	9.6E-01	5.4E-01
mitomycin	0.734	5.1E-09	2.6E-01	8.4E-02	8.4E-13	5.5E-01	5.6E-06	2.8E-05	2.9E-02	5.0E-05	5.7E-10	9.8E-01
niclosamide	0.687	6.9E-01	1.6E-02	1.8E-03	5.2E-08	3.8E-07	7.8E-11	1.2E-04	4.9E-04	5.7E-08	6.4E-09	8.5E-02
omacetaxine me	0.735	5.3E-08	2.2E-04	1.5E-08	1.3E-01	5.5E-03	8.6E-16	5.2E-01	1.2E-06	1.4E-07	8.8E-01	8.8E-01
paclitaxel	0.753	2.9E-08	8.6E-02	6.2E-05	1.4E-09	1.1E-10	2.9E-13	4.3E-08	2.9E-10	1.2E-05	1.1E-03	5.7E-01
PLX-4032	0.583	5.3E-01	8.8E-01	8.7E-02	3.7E-01	8.8E-08	6.8E-03	4.5E-01	1.8E-01	2.8E-02	9.8E-01	9.9E-01
prochlorperazine	0.609	2.6E-02	3.3E-02	8.4E-07	6.7E-01	7.5E-01	1.0E-10	9.5E-01	1.4E-01	2.3E-04	8.5E-02	5.7E-07
sirolimus	0.670	5.3E-01	6.0E-01	3.5E-01	1.8E-08	1.8E-07	1.6E-05	4.6E-05	6.5E-02	7.9E-02	4.1E-01	1.0E+00
sitagliptin	0.582	3.6E-01	6.8E-01	2.3E-02	5.7E-03	4.4E-02	4.5E-03	3.6E-02	3.7E-01	1.4E-01	4.5E-02	9.7E-01
teniposide	0.739	1.3E-03	2.1E-01	9.0E-01	3.6E-02	4.2E-01	6.1E-07	1.5E-01	1.3E-09	3.3E-03	8.1E-04	5.5E-06
topotecan	0.752	4.0E-14	7.0E-01	1.2E-05	2.6E-04	9.3E-02	3.3E-13	5.0E-04	2.5E-09	2.7E-06	3.5E-03	3.9E-04
trifluoperazine	0.659	7.2E-03	6.8E-03	1.0E+00	5.1E-01	5.2E-03	2.0E-05	7.0E-01	4.1E-03	3.4E-05	9.7E-01	5.4E-03
valdecoxib	0.708	1.5E-01	1.0E-01	5.3E-02	3.2E-01	8.9E-15	3.9E-06	1.3E-01	3.9E-07	9.8E-08	2.1E-01	6.3E-10
vincristine	0.788	1.6E-08	9.8E-01	3.5E-05	1.6E-08	1.7E-01	4.0E-11	1.6E-05	6.2E-06	5.7E-05	8.9E-02	1.0E+00
vorinostat	0.743	4.7E-01	4.2E-04	1.2E-04	1.1E-01	7.6E-05	5.2E-13	3.8E-01	6.7E-08	6.0E-08	1.3E-01	5.6E-08
% Dr.VAE ">"		61.5%	34.6%	57.7%	53.8%	53.8%	96.2%	46.2%	65.4%	88.5%	38.5%	42.3%
% Dr.VAE "≈"		38.5%	61.5%	30.8%	34.6%	34.6%	3.8%	50.0%	30.8%	11.5%	50.0%	38.5%
% Dr.VAE "<"		0.0%	3.8%	11.5%	11.5%	11.5%	0.0%	3.8%	3.8%	0.0%	11.5%	19.2%

Table S3: **Per-drug AUPR classification results.** Cross-validated test AUPR (area under PR curve) of our Dr.VAE to SSVAE and other classification models detailed for each evaluated drug. Methods including PCA and PertVAE are 2-step methods: (i) fit the unsupervised model, (ii) use latent representation to fit a standard classifier.

**AUPR**				baselines trained on pre-treatment $\mathbf{x}_1$			baselines trained on top 100 PCs of $\mathbf{x}_1$			baselines trained on PertVAE latent $\mathbf{z}_1$ of $\mathbf{x}_1$		
drug	**Dr.VAE (6h)**	**Dr.VAE w/ I**	SSVAE	RForest	RidgeLR	SVMrbf	RForest	RidgeLR	SVMrbf	Rforest	RidgeLR	SVMrbf
bortezomib	0.810	0.809	0.804	0.802	0.798	0.773	0.774	0.803	0.787	0.789	0.805	0.795
bosutinib	0.538	0.537	0.532	0.533	0.542	0.546	0.510	0.555	0.552	0.530	0.547	0.547
ciclosporin	0.380	0.378	0.384	0.361	0.371	0.389	0.351	0.363	0.375	0.342	0.377	0.373
clofarabine	0.823	0.822	0.820	0.811	0.813	0.814	0.795	0.816	0.803	0.806	0.820	0.815
dasatinib	0.830	0.829	0.827	0.820	0.823	0.832	0.823	0.829	0.841	0.816	0.825	0.828
decitabine	0.761	0.759	0.756	0.740	0.755	0.737	0.721	0.757	0.729	0.738	0.763	0.731
docetaxel	0.847	0.844	0.841	0.842	0.837	0.826	0.791	0.842	0.798	0.827	0.841	0.834
etoposide	0.758	0.753	0.758	0.742	0.759	0.768	0.734	0.755	0.736	0.741	0.753	0.762
fluvastatin	0.727	0.726	0.725	0.729	0.736	0.751	0.719	0.725	0.726	0.718	0.726	0.733
fulvestrant	0.371	0.370	0.366	0.399	0.387	0.379	0.349	0.394	0.370	0.363	0.380	0.379
gemcitabine	0.825	0.825	0.821	0.813	0.815	0.818	0.802	0.824	0.804	0.811	0.821	0.817
lovastatin	0.734	0.734	0.732	0.728	0.730	0.736	0.704	0.725	0.721	0.719	0.738	0.736
mitomycin	0.838	0.836	0.836	0.829	0.799	0.830	0.813	0.821	0.818	0.821	0.822	0.829
niclosamide	0.786	0.787	0.784	0.772	0.777	0.771	0.756	0.778	0.766	0.760	0.777	0.776
omacetaxine me	0.885	0.882	0.878	0.874	0.879	0.879	0.849	0.885	0.867	0.870	0.886	0.887
paclitaxel	0.848	0.847	0.844	0.842	0.833	0.823	0.819	0.839	0.809	0.833	0.844	0.840
PLX-4032	0.427	0.427	0.417	0.421	0.415	0.406	0.401	0.415	0.425	0.415	0.433	0.437
prochlorperazine	0.709	0.709	0.703	0.687	0.706	0.704	0.673	0.711	0.696	0.693	0.702	0.695
sirolimus	0.761	0.761	0.761	0.756	0.737	0.734	0.740	0.745	0.747	0.753	0.757	0.763
sitagliptin	0.396	0.395	0.400	0.388	0.400	0.404	0.354	0.402	0.364	0.376	0.392	0.404
teniposide	0.848	0.847	0.848	0.848	0.839	0.847	0.816	0.844	0.826	0.832	0.837	0.832
topotecan	0.859	0.856	0.857	0.847	0.852	0.849	0.835	0.852	0.833	0.842	0.855	0.842
trifluoperazine	0.351	0.348	0.342	0.357	0.355	0.357	0.338	0.357	0.366	0.327	0.344	0.350
valdecoxib	0.837	0.837	0.833	0.824	0.837	0.799	0.806	0.836	0.805	0.815	0.833	0.808
vincristine	0.856	0.853	0.855	0.848	0.844	0.853	0.840	0.851	0.843	0.848	0.854	0.859
vorinostat	0.865	0.865	0.860	0.859	0.863	0.849	0.840	0.864	0.849	0.850	0.862	0.844
MEAN	0.718	0.717	0.715	0.710	0.712	0.711	0.690	0.715	0.702	0.701	0.715	0.712

Table S4: **Per-drug statistical comparison of Dr.VAE to other evaluated methods by AUPR**. Statistical comparison of Dr.VAE to a set of evaluated baseline models on basis of their test area under precision-recall curve on 100 data splits. Shown is p-value of one-sided Wilcoxon Signed-Rank Test rejecting null hypothesis of "Dr.VAE performance is worse or no different from the compared model performance" in favor of alternative hypothesis "Dr.VAE mean performance is higher than the compared model". In Dr.VAE column, the mean test AUPR is shown.

**AUPR** drug	**Dr.VAE (6h)**	**Dr.VAE w/ I**	SSVAE	baselines trained on pre-treatment $\mathbf{x}_1$			baselines trained on top 100 PCs of $\mathbf{x}_1$			baselines trained on PertVAE latent $\mathbf{z}_1$ of $\mathbf{x}_1$		
				RForest	RidgeLR	SVMrbf	RForest	RidgeLR	SVMrbf	RForest	RidgeLR	SVMrbf
bortezomib	0.810	▼ 1.5E-03	▼ 9.4E-05	▼ 1.1E-04	▼ 6.1E-07	▼ 1.8E-16	▼ 1.7E-15	▼ 3.7E-06	▼ 7.6E-12	▼ 1.8E-12	▼ 3.0E-02	▼ 1.8E-07
bosutinib	0.538	▬ 1.8E-01	▼ 2.8E-02	▬ 1.4E-01	▬ 9.4E-01	▲ 9.9E-01	▼ 2.1E-07	▲ 1.0E+00	▲ 1.0E+00	▼ 1.2E-02	▲ 9.9E-01	▲ 9.9E-01
ciclosporin	0.380	▼ 7.0E-04	▬ 8.2E-01	▼ 2.6E-04	▼ 3.0E-02	▲ 9.5E-01	▼ 2.5E-06	▼ 4.9E-04	▬ 2.8E-01	▼ 2.1E-10	▬ 3.3E-01	▬ 7.6E-02
clofarabine	0.823	▼ 3.1E-05	▼ 2.9E-02	▼ 1.1E-09	▼ 1.0E-04	▼ 8.6E-08	▼ 2.1E-16	▼ 6.0E-05	▼ 4.4E-14	▼ 5.0E-14	▬ 5.3E-02	▼ 2.3E-06
dasatinib	0.830	▼ 6.5E-03	▼ 3.5E-02	▼ 6.1E-05	▼ 2.4E-03	▬ 9.4E-01	▼ 2.0E-02	▬ 5.5E-01	▲ 1.0E+00	▼ 2.3E-07	▼ 6.2E-03	▬ 1.5E-01
decitabine	0.761	▼ 4.3E-04	▼ 1.3E-02	▼ 1.7E-11	▼ 1.4E-02	▼ 3.9E-11	▼ 9.8E-17	▼ 6.6E-03	▼ 4.9E-15	▼ 7.1E-12	▬ 8.3E-01	▼ 1.7E-15
docetaxel	0.847	▼ 2.4E-07	▼ 3.3E-03	▬ 1.1E-01	▼ 3.6E-03	▼ 8.9E-10	▼ 2.2E-16	▼ 4.6E-02	▼ 1.0E-16	▼ 1.3E-07	▼ 1.2E-02	▼ 1.6E-04
etoposide	0.758	▼ 6.8E-08	▬ 2.8E-01	▼ 1.1E-05	▬ 5.9E-01	▲ 1.0E+00	▼ 6.7E-09	▬ 5.4E-02	▼ 4.7E-08	▼ 2.7E-06	▼ 7.7E-03	▬ 8.0E-01
fluvastatin	0.727	▼ 2.3E-03	▬ 1.4E-01	▬ 5.2E-01	▲ 1.0E+00	▲ 1.0E+00	▼ 5.7E-03	▬ 2.8E-01	▬ 3.5E-01	▼ 5.4E-03	▬ 3.8E-01	▬ 9.3E-01
fulvestrant	0.371	▬ 8.8E-02	▬ 2.3E-01	▲ 1.0E+00	▲ 9.7E-01	▬ 9.3E-01	▼ 2.1E-02	▲ 9.9E-01	▬ 7.5E-01	▬ 2.3E-01	▬ 8.5E-01	▬ 9.3E-01
gemcitabine	0.825	▬ 3.8E-01	▼ 8.9E-03	▼ 3.4E-08	▼ 1.7E-03	▼ 4.4E-04	▼ 1.6E-12	▬ 6.3E-01	▼ 2.5E-11	▼ 4.2E-09	▼ 8.4E-03	▼ 1.4E-04
lovastatin	0.734	▬ 3.3E-01	▬ 2.7E-01	▼ 4.4E-02	▼ 3.0E-02	▬ 6.0E-01	▼ 4.2E-11	▼ 6.9E-05	▼ 1.2E-04	▼ 9.8E-06	▬ 9.4E-01	▬ 6.8E-01
mitomycin	0.838	▼ 5.3E-09	▬ 8.2E-02	▼ 7.5E-06	▼ 1.1E-15	▼ 3.3E-04	▼ 1.1E-13	▼ 1.9E-12	▼ 7.1E-12	▼ 9.9E-12	▼ 2.9E-12	▼ 1.8E-05
niclosamide	0.786	▬ 9.1E-01	▬ 1.6E-01	▼ 1.2E-05	▼ 1.2E-03	▼ 6.5E-06	▼ 1.0E-12	▼ 6.3E-03	▼ 3.4E-09	▼ 4.1E-11	▼ 5.4E-04	▼ 1.3E-04
omacetaxine me	0.885	▼ 6.2E-07	▼ 9.3E-05	▼ 8.6E-06	▼ 9.4E-03	▼ 2.8E-03	▼ 1.5E-15	▬ 6.8E-01	▼ 1.5E-09	▼ 8.3E-08	▬ 9.0E-01	▬ 8.6E-01
paclitaxel	0.848	▼ 4.2E-04	▼ 2.5E-02	▼ 2.9E-04	▼ 1.1E-08	▼ 5.1E-14	▼ 6.9E-15	▼ 2.4E-07	▼ 2.1E-17	▼ 3.2E-08	▼ 1.3E-02	▼ 8.5E-04
PLX-4032	0.427	▬ 2.2E-01	▼ 6.9E-03	▬ 6.9E-02	▼ 2.0E-03	▼ 1.8E-06	▼ 1.8E-07	▼ 5.7E-04	▬ 3.7E-01	▼ 2.2E-03	▬ 9.2E-01	▲ 9.9E-01
prochlorperazine	0.709	▬ 1.3E-01	▼ 1.7E-02	▼ 4.7E-10	▬ 2.6E-01	▼ 4.8E-02	▼ 3.9E-14	▬ 8.0E-01	▼ 2.2E-05	▼ 1.6E-06	▼ 6.8E-03	▼ 8.6E-05
sirolimus	0.761	▬ 2.4E-01	▬ 4.2E-01	▬ 5.3E-02	▼ 7.8E-11	▼ 4.3E-13	▼ 1.1E-07	▼ 1.6E-07	▼ 2.8E-05	▼ 1.2E-03	▼ 2.6E-02	▬ 7.2E-01
sitagliptin	0.396	▬ 2.3E-01	▬ 4.9E-01	▬ 9.6E-02	▬ 4.7E-01	▬ 7.8E-01	▼ 1.9E-05	▬ 6.0E-01	▼ 4.4E-03	▼ 2.4E-02	▬ 1.7E-01	▬ 9.2E-01
teniposide	0.848	▼ 1.3E-04	▬ 3.4E-01	▬ 4.9E-01	▼ 2.3E-03	▬ 2.6E-01	▼ 5.5E-11	▼ 4.5E-02	▼ 3.3E-11	▼ 2.0E-07	▼ 2.2E-06	▼ 1.1E-08
topotecan	0.859	▼ 5.6E-12	▬ 1.1E-01	▼ 3.1E-09	▼ 7.6E-05	▼ 1.6E-06	▼ 7.7E-15	▼ 1.7E-04	▼ 4.1E-16	▼ 2.0E-11	▼ 3.7E-03	▼ 1.2E-11
trifluoperazine	0.351	▼ 7.2E-03	▼ 2.8E-02	▬ 8.7E-01	▬ 6.5E-01	▬ 8.9E-01	▼ 1.5E-02	▬ 7.8E-01	▲ 1.0E+00	▼ 1.5E-04	▬ 1.4E-01	▬ 4.8E-01
valdecoxib	0.837	▬ 5.1E-01	▼ 1.2E-02	▼ 7.7E-10	▬ 3.3E-01	▼ 1.9E-17	▼ 5.6E-15	▬ 1.8E-01	▼ 4.9E-15	▼ 1.7E-13	▼ 1.0E-02	▼ 8.5E-17
vincristine	0.856	▼ 3.6E-12	▬ 4.6E-01	▼ 1.9E-05	▼ 2.5E-07	▼ 3.5E-02	▼ 2.0E-10	▼ 1.7E-02	▼ 2.8E-07	▼ 1.8E-05	▬ 1.1E-01	▲ 9.6E-01
vorinostat	0.865	▬ 7.3E-01	▼ 1.8E-03	▼ 7.2E-04	▬ 1.8E-01	▼ 7.9E-09	▼ 3.2E-15	▬ 4.7E-01	▼ 1.8E-10	▼ 2.1E-10	▬ 6.5E-02	▼ 2.0E-12
% Dr.VAE ">"		57.7%	53.8%	65.4%	65.4%	61.5%	100.0%	53.8%	73.1%	96.2%	50.0%	50.0%
% Dr.VAE "≈"		42.3%	46.2%	30.8%	26.9%	23.1%	0.0%	38.5%	15.4%	3.8%	46.2%	38.5%
% Dr.VAE "<"		0.0%	0.0%	3.8%	7.7%	15.4%	0.0%	7.7%	11.5%	0.0%	3.8%	11.5%

Table S5: **Overall statistical comparison of Dr.VAE to other evaluated methods.** Wilcoxon signed-rank test p-values that the performance of Dr.VAE is overall better (greater) than that of a compared method in terms of their test AUROC and AUPR, respectively. The Wilcoxon paired test is conducted on the methods' average per-drug performance on the set of tested 26 drugs, i.e. comparing corresponding columns in Table S1 and Table S3, for AUROC and AUPR measure, respectively.

compared method		p-value that Dr.VAE performance is greater			
		uncorrected p-values		Bonferroni corrected p-vals	
		AUROC	AUPR	AUROC	AUPR
	DrVAE w/ I	9.36e-06	2.05e-05	1.03e-04	2.26e-04
	SSVAE	1.97e-03	1.48e-04	2.17e-02	1.63e-03
baselines trained on pre-treatment $\mathbf{x}_1$	RForest	1.97e-03	1.80e-04	2.17e-02	1.98e-03
	RidgeLR	2.18e-04	1.42e-03	2.40e-03	1.56e-02
	SVMrbf	4.19e-04	1.92e-02	4.61e-03	2.11e-01
baselines trained on top 100 PCs of $\mathbf{x}_1$	RForest	4.15e-06	4.15e-06	4.57e-05	4.57e-05
	RidgeLR	1.54e-03	1.08e-02	1.69e-02	1.19e-01
	SVMrbf	2.55e-05	7.32e-05	2.81e-04	8.05e-04
baselines trained on PertVAE latent $\mathbf{z}_1$ of $\mathbf{x}_1$	RForest	4.15e-06	4.15e-06	4.57e-05	4.57e-05
	RidgeLR	2.71e-03	8.20e-03	2.98e-02	9.02e-02
	SVMrbf	2.31e-02	2.17e-02	2.54e-01	2.39e-01

Table S6: **Dataset summarization.** For each from the selected set of 26 FDA-approved drugs, this table shows the number of cell lines tested in CMap-L1000v1 for drug-induced perturbation effects on gene expression, the total number of extracted control-perturbation pairs including biological replicates, as well as number of these cell lines for which drug sensitivity was matched and retrieved from CTRPv2. Next, shown is effect-to-replicate variance ratio that quantifies signal-to-noise strength in the perturbation experiments. The last two columns show the number of drug-response-labeled samples (out of 927 total cell lines) and the ratio of positive responders in CTRPv2 drug sensitivity data set.

	**CMap-L1000v1 perturbation data set**								**CTRPv2 sensitivity d.s.**	
drug	labeled pairs	labeled unique CLs	unlabeled pairs	unlabeled unique CLs	total pairs	total unique CLs	effect/rep. variance ratio	effect/rep. silhouette score	number of labeled CLs	responder %
bortezomib	97	39	29	12	126	51	0.512	0.046	824	67.60%
bosutinib	26	7	14	6	40	13	0.209	-0.023	823	24.54%
ciclosporin	219	36	49	13	268	49	0.226	-0.026	808	20.67%
clofarabine	27	7	5	2	32	9	0.248	-0.004	854	58.43%
dasatinib	43	11	10	3	53	14	0.266	0.011	845	59.05%
decitabine	23	7	26	6	49	13	0.142	-0.044	849	25.91%
docetaxel	22	2	34	7	56	9	0.245	-0.046	422	64.69%
etoposide	25	6	13	5	38	11	0.356	0.018	840	26.90%
fluvastatin	44	7	16	6	60	13	0.215	-0.060	820	59.02%
fulvestrant	23	2	33	7	56	9	0.141	-0.022	206	25.24%
gemcitabine	74	28	50	23	124	51	0.366	-0.024	777	59.07%
lovastatin	66	9	33	7	99	16	0.180	-0.008	849	59.95%
mitomycin	64	7	11	2	75	9	0.237	0.008	838	60.14%
niclosamide	146	39	48	14	194	53	0.415	0.015	830	63.73%
omacetaxine mep	19	4	22	6	41	10	0.645	0.041	625	74.88%
paclitaxel	90	9	29	3	119	12	0.175	-0.017	827	64.81%
PLX-4032	138	41	37	13	175	54	0.356	-0.011	820	25.37%
prochlorperazine	55	7	6	2	61	9	0.049	-0.035	823	60.63%
sirolimus	330	44	87	16	417	60	0.262	-0.024	852	58.33%
sitagliptin	13	4	19	5	32	9	0.254	0.003	212	26.42%
teniposide	67	27	54	23	121	50	0.415	-0.017	410	63.17%
topotecan	42	6	8	2	50	8	0.494	0.118	855	64.56%
trifluoperazine	164	35	55	20	219	55	0.339	-0.035	782	21.10%
valdecoxib	91	36	28	13	119	49	0.373	-0.019	803	63.51%
vincristine	42	7	14	2	56	9	0.198	-0.007	845	60.83%
vorinostat	98	40	47	15	145	55	0.647	0.105	825	67.03%
MEAN	78.77	17.96	29.88	8.96	108.65	26.92	0.306	-0.002	740.92	50.98%

Table S7: **Post-treatment expression prediction results.** Shown is prediction RMSE of full Dr.VAE model on post-treatment latent representation $\mathbf{z}_2$ and post-treatment gene expression $\mathbf{x}_2$ computed on training and validation sets, and the Δ improvement of full Dr.VAE over Dr.VAE with an identity function instead of learned perturbation function (denoted "Dr.VAE w/ I" in the main text) in these measures. Pearson correlation of these Δ improvements to data set statistics are shown in Table 1 of the main text.

**RMSE**	*training set*				*validation set*				CMap-L1000v1 stats	
drug	RMSE of predicted $\mathbf{z}_2$	Δ RMSE over $\mathbf{z}_2$ w.r.t. $\mathbf{I}$	RMSE of predicted $\mathbf{x}_2$	Δ RMSE over $\mathbf{x}_2$ w.r.t. $\mathbf{I}$	RMSE of predicted $\mathbf{z}_2$	Δ RMSE over $\mathbf{z}_2$ w.r.t. $\mathbf{I}$	RMSE of predicted $\mathbf{x}_2$	Δ RMSE over $\mathbf{x}_2$ w.r.t. $\mathbf{I}$	effect/rep. variance ratio	number of unique CLs in CMap
bortezomib	0.248	0.090	0.511	0.014	0.443	0.025	0.608	0.008	0.512	51
bosutinib	0.297	0.026	0.513	0.011	0.529	-0.075	0.675	-0.004	0.209	13
ciclosporin	0.326	0.039	0.525	0.005	0.455	-0.023	0.592	0.001	0.226	49
clofarabine	0.316	0.025	0.467	0.011	0.490	-0.040	0.622	-0.003	0.248	9
dasatinib	0.289	0.030	0.492	0.010	0.495	-0.036	0.640	0.001	0.266	14
decitabine	0.281	0.012	0.485	0.007	0.462	-0.045	0.613	-0.003	0.142	13
docetaxel	0.316	-0.013	0.518	0.005	0.503	-0.114	0.618	-0.003	0.245	9
etoposide	0.305	0.019	0.524	0.010	0.530	-0.077	0.687	-0.003	0.356	11
fluvastatin	0.288	0.004	0.530	0.005	0.448	-0.057	0.641	-0.002	0.215	13
fulvestrant	0.302	-0.007	0.507	0.004	0.406	-0.044	0.603	-0.002	0.141	9
gemcitabine	0.248	0.068	0.514	0.008	0.478	-0.009	0.618	-0.003	0.366	51
lovastatin	0.304	0.013	0.515	0.004	0.489	-0.065	0.634	-0.001	0.180	16
mitomycin	0.342	0.005	0.500	0.008	0.500	-0.070	0.604	-0.001	0.237	9
niclosamide	0.287	0.056	0.550	0.009	0.436	-0.009	0.636	0.004	0.415	53
omacetaxine mep	0.283	0.089	0.531	0.031	0.522	-0.001	0.753	0.008	0.645	10
paclitaxel	0.316	0.000	0.509	0.004	0.505	-0.118	0.625	-0.002	0.175	12
PLX-4032	0.270	0.051	0.538	0.007	0.429	-0.013	0.619	0.002	0.356	54
prochlorperazine	0.303	-0.004	0.501	0.005	0.417	-0.065	0.593	-0.001	0.049	9
sirolimus	0.321	0.040	0.559	0.005	0.463	-0.031	0.621	0.002	0.262	60
sitagliptin	0.306	0.006	0.448	0.007	0.498	-0.060	0.581	-0.004	0.254	9
teniposide	0.240	0.083	0.506	0.012	0.439	0.011	0.610	0.002	0.415	50
topotecan	0.363	0.059	0.570	0.031	0.663	-0.072	0.774	-0.004	0.494	8
trifluoperazine	0.299	0.032	0.535	0.005	0.467	-0.046	0.611	-0.002	0.339	55
valdecoxib	0.239	0.065	0.514	0.007	0.447	-0.004	0.611	-0.001	0.373	49
vincristine	0.347	-0.001	0.505	0.008	0.519	-0.095	0.625	-0.003	0.198	9
vorinostat	0.256	0.111	0.511	0.018	0.468	0.040	0.617	0.013	0.647	55
MEAN	0.296	0.035	0.514	0.010	0.481	-0.042	0.632	0.000	0.306	26.92

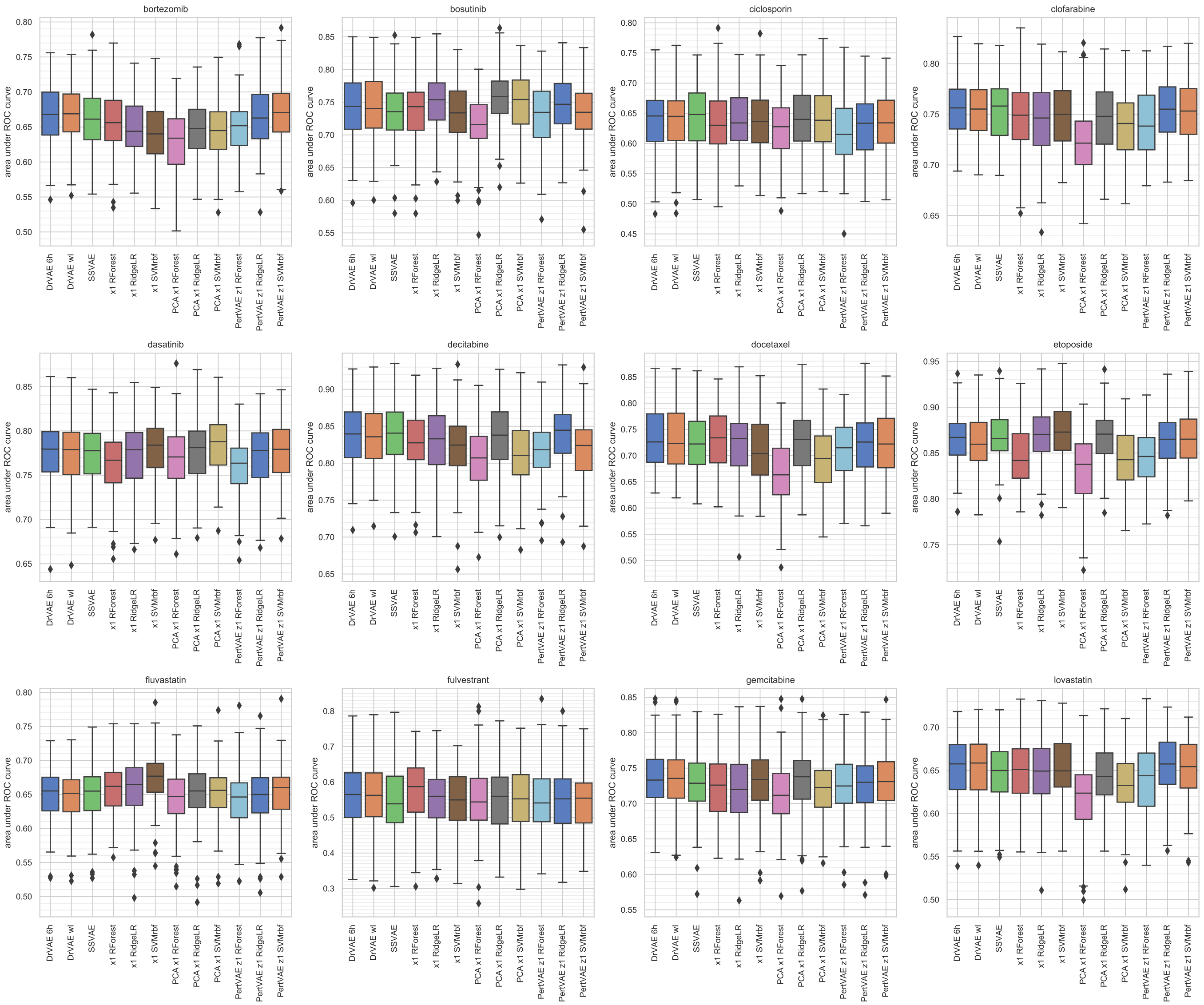

Figure S3: **Boxplots of test AUROC in 100 data splits.** For each of 26 tested drugs we show the distribution of test set area under ROC curve of 12 evaluated methods in 20 times repeated 5-fold CV. (continues on the next page)

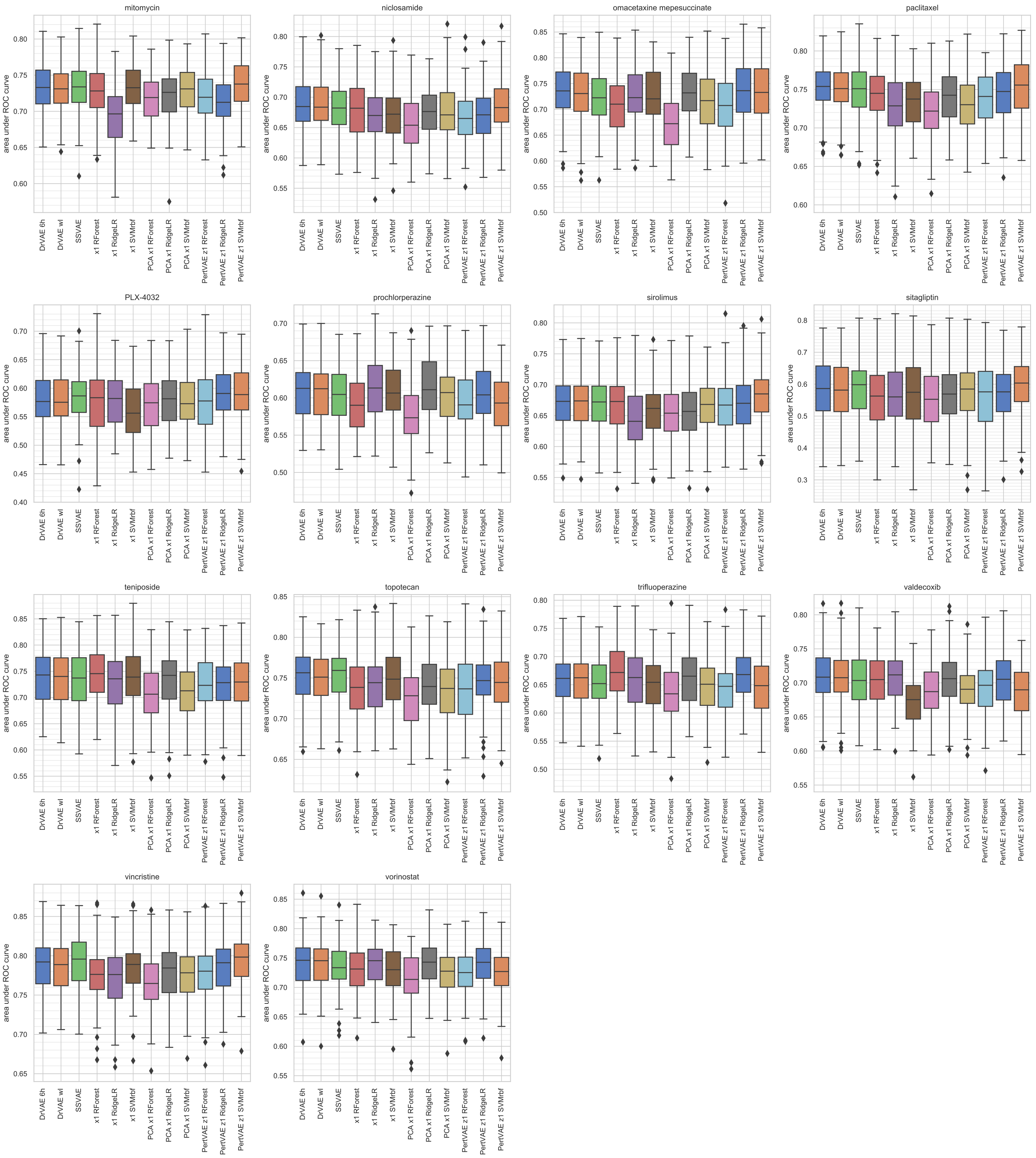

Figure S3: **Boxplots of test AUROC in 100 data splits.** (continued)

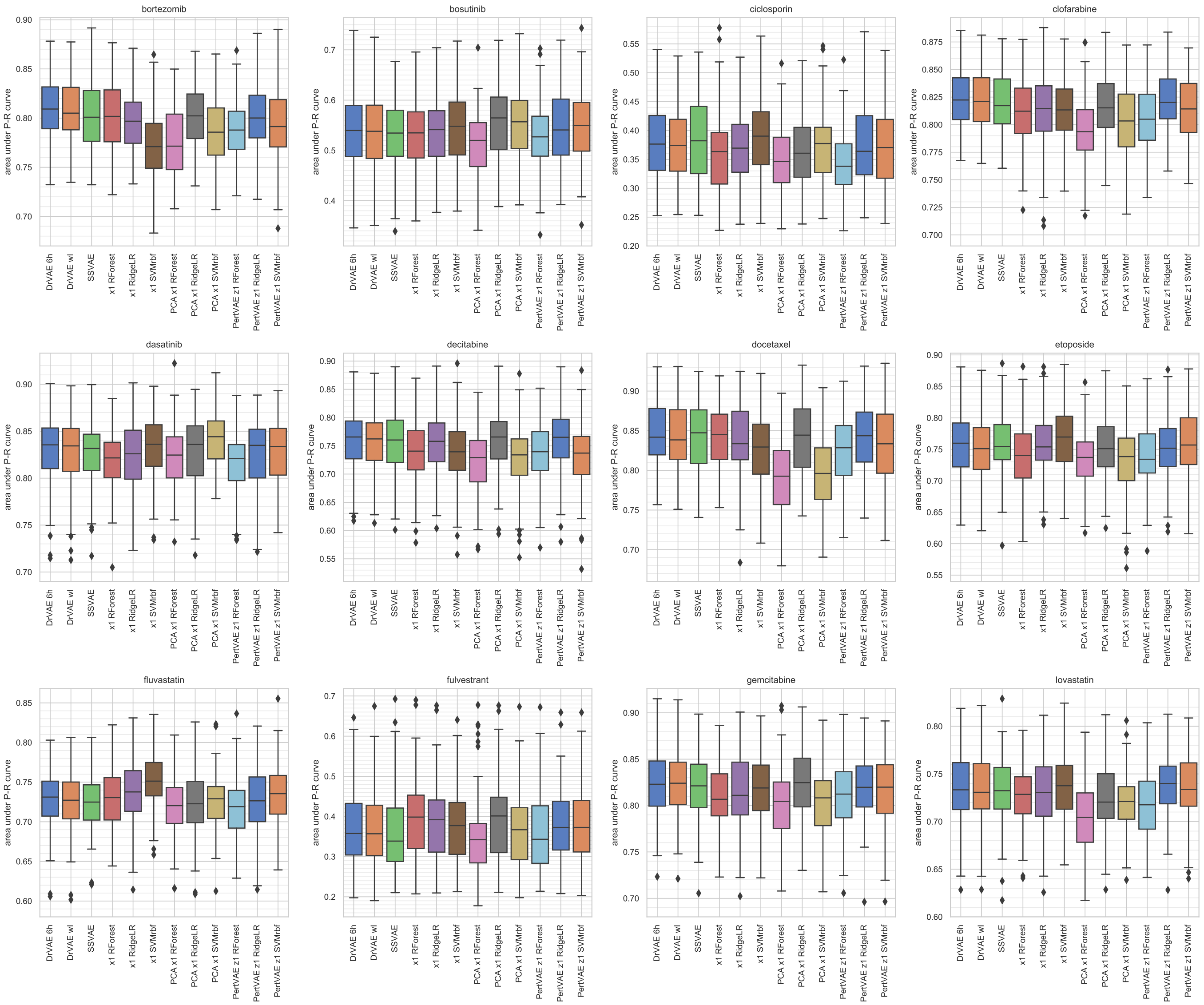

Figure S4: **Boxplots of test AUPR in 100 data splits.** For each of 26 tested drugs we show the distribution of test set area under Precision-Recall curve of 12 evaluated methods in 20 times repeated 5-fold CV. (continues on the next page)

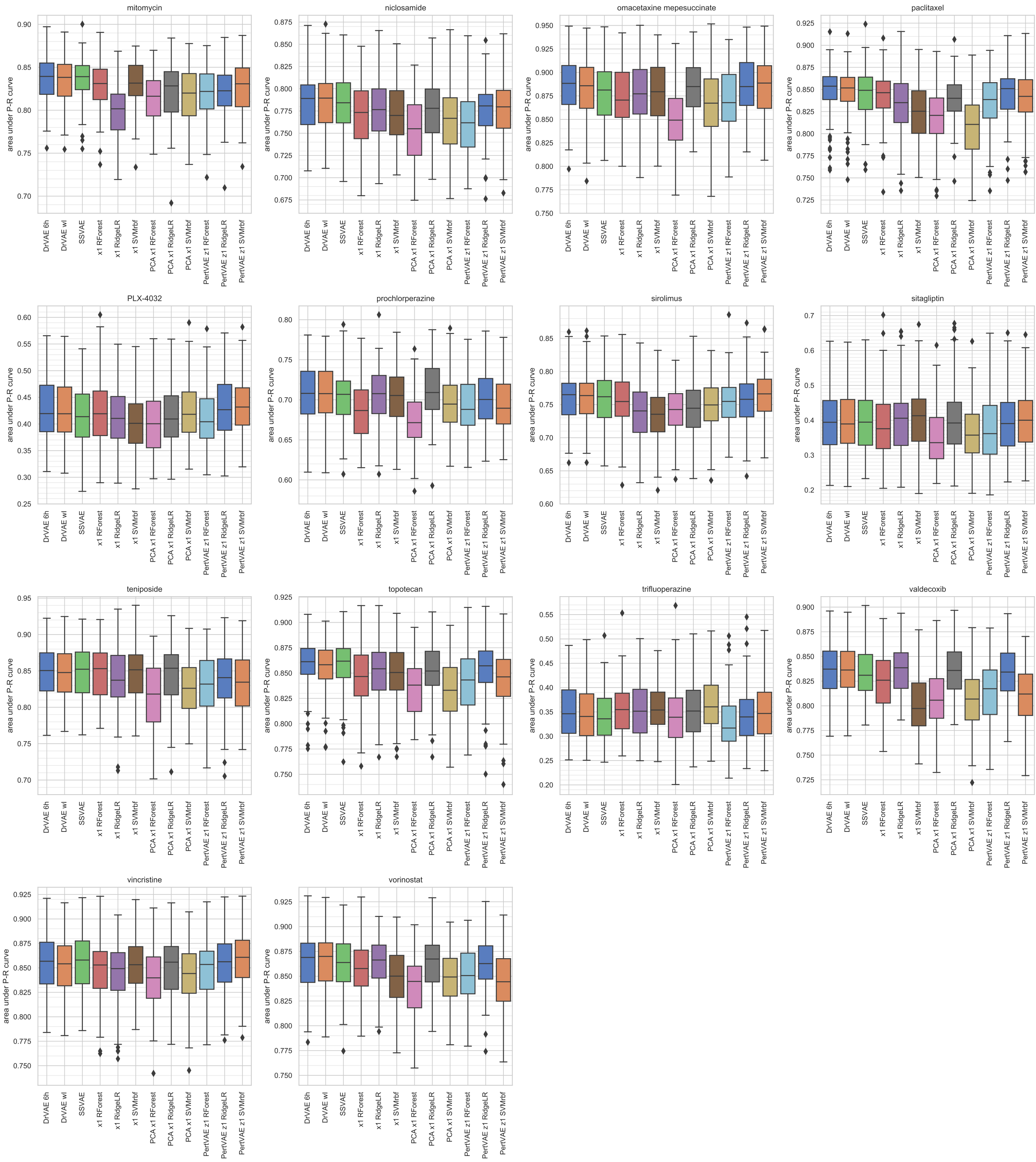

Figure S4: **Boxplots of test AUPR in 100 data splits.** (continued)
